# Genome-Wide Analysis of Transposable Elements and Satellite DNAs in *Spinacia* Species to Shed Light on Their Roles in Sex Chromosome Evolution

**DOI:** 10.3389/fpls.2020.575462

**Published:** 2021-01-14

**Authors:** Ning Li, Xiaoyue Li, Jian Zhou, Li’ang Yu, Shufen Li, Yulan Zhang, Ruiyun Qin, Wujun Gao, Chuanliang Deng

**Affiliations:** ^1^College of Life Sciences, Henan Normal University, Xinxiang, China; ^2^Department of Plant Biology, University of Illinois at Urbana-Champaign, Champaign, IL, United States

**Keywords:** repetitive DNA sequences, rDNA, homomorphic sex chromosome, heteromorphic sex chromosome, long terminal repeat retroelements, next generation sequencing

## Abstract

Sex chromosome evolution has mostly been studied in species with heteromorphic sex chromosomes. The *Spinacia* genus serves as an ideal model for investigating evolutionary mechanisms underlying the transition from homomorphic to heteromorphic sex chromosomes. Among evolutionary factors, repetitive sequences play multiple roles in sex chromosome evolution while their forces have not been fully explored in *Spinacia* species. Here, we identified major repetitive sequence classes in male and female genomes of *Spinacia* species and their ancestral relative sugar beet to elucidate the evolutionary processes of sex chromosome evolution using next-generation sequencing (NGS) data. Comparative analysis revealed that the repeat elements of *Spinacia* species are considerably higher than of sugar beet, especially the *Ty3/Gypsy* and *Ty1/Copia* retrotransposons. The long terminal repeat retroelements (LTR) Angela, Athila, and Ogre may be accounted for the higher proportion of repeats in the spinach genome. Comparison of the repeats proportion between female and male genomes of three *Spinacia* species indicated the different representation in *Spinacia tetrandra* samples but not in the *S. oleracea* or *S. turkestanica* samples. From these results, we speculated that emergence of repetitive DNA sequences may correlate the formation of sex chromosome and the transition from homomorphic sex chromosomes to heteromorphic sex chromosomes as heteromorphic sex chromosomes exclusively existed in *Spinacia tetrandra*. Three novel sugar beet-specific satellites were identified and confirmed by fluorescence *in situ* hybridization (FISH); six out of eight new spinach-specific satellites were mapped to the short arm of sex chromosomes. A total of 141 copies of SolSat01-171-s were found in the sex determination region (SDR). Thus, the accumulation of satellite DNA on the short arm of chromosome 1 may be involved in the sex chromosome evolution in *Spinacia* species. Our study provides a fundamental resource for understanding repeat sequences in *Spinacia* species and their roles in sex chromosome evolution.

## Introduction

The *Spinacia* genus belongs to family Chenopodiaceae. It includes cultivated spinach (*S. oleracea* L.) and two wild relatives (*S. turkestanica* Ilj. and *S. tetrandra* Stev.). The cultivated spinach genome was approximately 989 Mb (Arumuganathan and Earle, 1991). The direct progenitor of cultivated spinach is likely *S. turkestanica*, which was generally more closely related to the cultivated spinach than *S. tetrandra* (Xu et al., 2017). The three species of *Spinacia* are dioecious and occasionally monoecious, which is controlled by a pair of sex chromosomes X/Y (Onodera et al., 2008). The sex chromosomes of all *S. oleracea* accessions and all *S. turkestanica* accessions are homomorphic. However, sex chromosomes of two *S. tetrandra* accessions are homomorphic and the other three accessions are heteromorphic (Fujito et al., 2015). Both homomorphic and heteromorphic sex chromosomes evolved from a common ancestral homologous autosomes. For the homomorphic sex chromosomes in *Spinacia* genus, the Y chromosome was predicted to have diverged recently with its counterpart X chromosome (0.40 ± 0.08 Mya); the homomorphic sex chromosomes and heteromorphic sex chromosomes may have diverged approximately 5.7 Mya (Okazaki et al., 2019). The co-existence of two types of sex chromosomes under *Spinacia* makes it to be a good model for investigating the evolution of heteromorphic sex chromosomes from ancestral homomorphic sex chromosomes.

Species with nine pairs of chromosomes are prevalent in Chenopodiaceae. Molecular phylogenetic evidence revealed an independent dysploid chromosome loss in two lineages of Chenopodiaceae [one is *Spinacia* (2n = 2x = 12)]. The monoecious *Blitum* (2n = 2x = 18) is relative to *Spinacia* in Chenopodiaceae, indicating dysploidy loss happened during *Spinacia* evolution (Kühn et al., 1993; Fuentes-Bazan et al., 2012a, b). *Spinacia* diverged with *Blitum* was approximately 15.4 Mya (based on *rbcL*) and 17.4 Mya (based on *atpB-rbcL*) (Kadereit et al., 2010).

Sugar beet (*Beta vulgaris*, 2n = 2x = 18) is an important crop with hermaphroditic flowers in the Chenopodiaceae with an estimated genome size of 567 Mb, of which approximately 42.3% are repetitive sequences (Paesold et al., 2012; Dohm et al., 2014; Kowar et al., 2016). Many repetitive sequences, such as satellite DNAs, transposable elements, retrotransposons, and DNA transposons, have been fully investigated in the sugar beet genome (Weber and Schmidt, 2009; Wenke et al., 2009; Weber et al., 2010; Zakrzewski et al., 2010, 2014; Menzel et al., 2012; Wollrab et al., 2012; Heitkam et al., 2014; Schwichtenberg et al., 2016). The divergence between *Arabidopsis* and the common ancestor of spinach and sugar beet occurred following an ancient triplication event (g), while sugar beet may have diverged from spinach approximately 38.4 Mya (Takahata et al., 2016; Xu et al., 2017).

Eukaryotic genomes contain abundant repetitive DNA, but the relationship between repeat composition and genome size remains unclear. Research on plant sex chromosome evolution has mostly focused on species with heteromorphic sex chromosomes, such as *Cannabis sativa* (Sakamoto et al., 2000), *Hippophae rhamnoides* (Puterova et al., 2017), *Coccinia grandis* (Sousa et al., 2016), *Silene latifolia*, and *Rumex acetosa* (Vyskot and Hobza, 2004). Repetitive sequences are important for sex chromosome evolution, and are involved in recombination suppression, heterochromatization, sex chromosomes differentiation in structure, Y degeneration, and X dosage compensation (Hobza et al., 2015; Li et al., 2016). In spinach, 74.4% of the genome is repetitive sequences (Xu et al., 2017) and the region around the male-determining locus is highly repetitive (Kudoh et al., 2018). The repetitive components of the *S. oleracea* L genome was annotated in our previous study, where we found that Ogre/Tat lineage and two sex chromosome-specific satellites DNAs may be involved in sex chromosome evolution (Li et al., 2019). However, the role of repetitive sequences in sex chromosome evolution of *Spinacia* has not been fully investigated.

Graph-based clustering of sequence reads is a novel method of identifying repetitive elements following next-generation sequencing (NGS) (Novak et al., 2014). RepeatExplorer is a computational pipeline to perform *de novo* repeat elements identification using a graph-based sequence clustering algorithm (Novak et al., 2013). This pipeline has been used to perform repetitive sequence classification and estimate sequence similarity among individual genomes (Feng et al., 2017; Gaiero et al., 2019; Liu et al., 2019; Pamponet et al., 2019).

This study aimed to elucidate variations of repetitive sequences regarding distribution of male vs. female genomes of three *Spinacia* species, and to compare genomic repeat DNA sequences of the *Spinacia* species with their relative sugar beet; as such, the evolutionary processes shaping the sex chromosome. Global identification and classification of repeats elements from male and female *Spinacia* species were conducted to characterize the distribution of elements and relations to sex chromosomes evolution. We also surveyed the potential roles of repetitive sequences in genome expansion in *Spinacia* wild relatives, which might be related to the emergence of heteromorphic sex chromosomes. Our study provides a fundamental resource for understanding repeat sequences in *Spinacia* species and their roles in sex chromosome evolution.

## Materials and Methods

### Plant Materials

The seeds of three species of *Spinacia*, including *S. oleracea* L., *S. turkestanica* Ilj., and *S. tetrandra* Stev., were obtained from the U.S. National Plant Germplasm System (NPGS)^1^. Plant specimen information was deposited in NPGS. The accession numbers of the plant materials used in our study are PI 527332 (*S. oleracea* L.), PI 664498 (*S. oleracea* L.), PI 478393 (*S. oleracea* L.), PI 647862 (*S. turkestanica* Ilj.), PI 604792 (*S. turkestanica* Ilj.), PI 494751 (*S. turkestanica* Ilj.), PI 647861 (*S. tetrandra* Stev.), PI 677114 (*S. tetrandra* Stev.), and PI 608712 (*S. tetrandra* Stev.). The plant name and origin of plant specimen are listed in Supplementary Table 1.

Samples were planted in a garden field of Henan Normal University. The fresh leaves from male and female plants from each species were collected in flowering phase and DNA was extracted using traditional cetyl trimethylammonium bromide method (Rogers and Bendich, 1988).

### Next-Generation Sequencing

We prepared three DNA aliquots (10 μL; ∼2 μg/μL) of each sample in parallel [samples 1–17; Sp-OL-M-1 (PI 527332), Sp-OL-F-1 (PI 527332), Sp-OL-M-2 (PI 664498), Sp-OL-F-2 (PI 664498), Sp-OL-M-3 (PI 478393), and Sp-OL-F-3 (PI 478393) from *S. oleracea*; Sp-TU-M-1 (PI 647862), Sp-TU-F-1 (PI 647862), Sp-TU-M-2 (PI 604792), Sp-TU-F-2 (PI 604792), Sp-TU-M-3 (PI 494751), and Sp-TU-F-3 (PI 494751) from *S. turkestanica*; Sp-TE-M-1 (PI 647861), Sp-TE-F-1 (PI 647861), Sp-TE-M-2 (PI 677114), Sp-TE-M-3 (PI 608712), and Sp-TE-F-3 (PI 608712) from *S. tetrandra*]. Paired-end libraries with insert sizes of 400 bp were constructed using TruSeq Library Construction Kit (Illumina^®^) and sequenced by an Illumina HiSeq4000 platform (LC⋅BioTech, Hangzhou, China). Then 20 Gb raw data sequencing reads were generated and trimmed using the FASTX Toolkit, and merged using the “fastqjoin” software with default Settings.

### Repetitive DNA Characterization and Identification

RepeatExplorer pipeline^2^ (Novak et al., 2010, 2013) was used to detect and characterize the repetitive DNA families based on the NGS data of spinach and raw data (SRR952972) of the sugar beet genome from NCBI database (Dohm et al., 2014). Clustered read repeats were identified by *De novo* Cluster analysis using a graph-based approach.

### Satellite DNA Identification

The TAREAN tool in RepeatExplorer was used to identify satellite DNA (Novak et al., 2017). Satellites were automatically detected based on the parameters “Connected component index (*C*)” and “Pair completeness index (*P*).” The identified satellites with *C* and *P* index close to one were defined as high-confidence satellites and used to develop FISH probes.

### Fluorescence *in situ* Hybridization and Microscopy Imaging

Oligonucleotide probes were designed from DNA elements identified by TAREAN tool. For monomers of satellite DNA, 50 bp nucleotides was randomly selected and labeled directly with Texas Red-X (Invitrogen, Shanghai, China) during synthesis. 45S rDNA was labeled with Chroma Tide Alexa Fluor 488-5-dUTP (Invitrogen) to identify corresponded chromosomes.

Chromosome spreads from root tips were prepared as described from previous studies (Deng et al., 2012). Briefly, selected slides were cross-linked under UV for 2 min. The probe solution (20 ng/μL of each probe in 2 × saline-sodium citrate and 1 × Tris–ethylenediaminetetraacetic acid buffer) was denatured in boiling water for 5 min and kept on ice before use. Six μL of the denatured probe solution was added to each slide, heated for 5 min at 100°C, and left overnight at 55°C in a humid chamber. The slides were washed in 2 × saline-sodium citrate, mounted using the Vectashield mounting medium (containing 1.5 μg/mL 4’, 6-diamidino-2-phenylindole; Vector Laboratories, Burlingame, United States). FISH images were captured by ANDOR CCD under an Olympus BX63 fluorescence microscope.

### Statistical Analysis

Each sample was validated with three replications. Multiple comparisons were performed to evaluate the difference between data groups by using SPSS software. Data were analyzed using least significance difference (LSD), and the significance level is 0.05.

## Results

### Repeat Proportion in Spinach and Sugar Beet Genome

**FIGURE 1:**
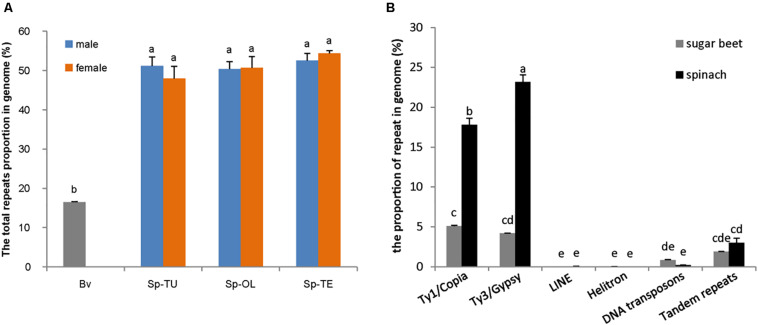
Comparison of repetitive sequence between spinach and sugar beet genome. **(A)** The total repeats proportion in spinach and sugar beet genome; Bv, sugar beet; Sp-TU, *S. turkestanica*; Sp-OL, *S. oleracea*; Sp-TE, *S. tetrandra.* **(B)** The repeat proportion in spinach and sugar beet genome. “a, b, c, d, e” means significance level by multiple comparison, *p* < 0.05.

NGS generated 20 Gb of raw sequencing reads (Illumina 300 bp paired end) for each sample from *S. oleracea*, *S. turkestanica*, and *S. tetrandra* with an average 44.43% of guanine-cytosine (GC) content (Supplementary Table 2). The raw reads (SRR952972) of sugar beet genome were downloaded from NCBI^3^. For gragh-based clustering of reads, 800–1,000 Mb subsets were analyzed using RepeatExplorer. We estimated the repeat proportions of the genome of *Spinacia* species and sugar beet through comparative clustering in Repeat Explorer. As a result, the proportions of combined repeats identified in each species ranged from 16.45 to 54.34% of their respective estimated genome size (Table 1 and Supplementary Table 3). Interestingly, the proportion of repeat in the spinach genome is much higher than that in sugar beet (Table 1 and Figure 1A). The repeats identified were further classified by cluster shape and sequence similarities. As shown in Table 1 and Figure 1B, the long terminal repeat (LTR) retrotransposons *Ty3/Gypsy* and *Ty1/Copia* are the main forms of the repetitive elements in spinach (23.31 and 17.69%, respectively) and sugar beet (4.17 and 5.17%, respectively); tandem repeats, including rDNA and satellites, make up nearly 3.02 and 1.91% of the spinach genome and sugar beet genome, respectively. Nevertheless, DNA transposons only make up 2.12 and 0.84%, respectively. The repeat elements with the lowest proportions are LINE and Helitron, which are less than 0.1% of the spinach and sugar beet genomes. Among these repeats, the proportions of *Ty3/Gypsy* and *Ty1/Copia* were significantly enriched in the spinach genome, compared with that of sugar beet. Thus, *Ty3/Gypsy* and *Ty1/Copia* may be accounted for the increasing proportion of repeats in the spinach genome.

**TABLE 1:** Classification of repetitive sequences identified in the spinach and sugar beet genomes.

Repeats		Lineage/class	*Beta_vulgaris*			Sp-TU-M			Sp-TU-F			Sp-OL-M		
			Bv_1 (%)	Bv_9 (%)	Bv (%)	PI647862-M (%)	PI604792-M (%)	PI494751-M (%)	PI647862-F (%)	PI604792-F (%)	PI494751-F (%)	PI527332-M (%)	PI664498-M (%)	PI478393-M (%)
LTR retroelements	Ty1/Copia	Ale	0.160	0.140	0.130	0.170	0.200	0.240	0.110	0.160	0.240	0.210	0.140	0.280
		Angela	0.360	0.340	0.350	19.340	18.020	15.880	21.60	20.220	16.520	16.780	17.790	14.430
		Bianca	0.180	0.220	0.190	0.000	0.010	0.000	0.020	0.030	0.000	0.000	0.010	0.000
		SIRE	3.740	3.580	3.760	0.930	0.660	0.250	0.800	0.610	0.230	0.390	0.750	0.220
		TAR	0.350	0.340	0.370	0.290	0.280	0.240	0.290	0.360	0.090	0.190	0.280	0.230
		Tork	0.440	0.320	0.380	0.010	0.020	0.010	0.000	0.000	0.020	0.000	0.020	0.010
		Others	0.000	0.000	0.000	0.250	0.430	0.060	0.000	0.000	0.350	0.030	0.000	0.280
		**Total**	**5.230**	**4.940**	**5.180**	**20.990**	**19.620**	**16.680**	**22.820**	**21.380**	**17.450**	**17.60**	**18.990**	**15.450**
	Ty3/Gypsy	Athila	0.650	0.220	0.280	1.440	1.230	1.160	1.690	1.220	1.280	1.780	1.450	1.370
		Ogre	0.000	0.000	0.000	15.630	17.270	17.190	10.550	13.740	17.510	18.330	14.780	16.910
		Retand	1.310	1.110	1.160	0.000	4.280	6.440	0.000	3.820	5.410	0.000	3.320	7.610
		CRM	0.980	0.940	1.030	0.360	0.330	0.260	0.300	0.380	0.270	0.300	0.320	0.230
		Galadriel	0.000	0.000	0.000	0.070	0.020	0.000	0.020	0.000	0.110	0.130	0.080	0.130
		Tekay	1.280	1.850	1.710	2.020	1.920	1.530	1.840	2.050	1.560	1.640	1.960	1.310
		**Total**	**4.220**	**4.120**	**4.180**	**19.520**	**25.050**	**26.580**	**14.400**	**21.210**	**26.140**	**22.180**	**21.910**	**27.560**
Other	LINE		0.000	0.000	0.000	0.120	0.070	0.000	0.050	0.160	0.100	0.060	0.100	0.000
	Helitron		0.050	0.020	0.020	0.010	0.000	0.000	0.000	0.000	0.000	0.000	0.000	0.010
	DNA transposons	EnSpm_CACTA	0.810	0.760	0.800	0.250	0.220	0.180	0.160	0.270	0.180	0.160	0.230	0.160
		MuDR_Mutator	0.150	0.010	0.000	0.050	0.010	0.000	0.010	0.030	0.000	0.020	0.010	0.030
		Tc1_Mariner	0.000	0.000	0.000	0.050	0.030	0.020	0.030	0.040	0.000	0.020	0.030	0.000
		**Total**	**0.960**	**0.770**	**0.800**	**0.350**	**0.260**	**0.200**	**0.200**	**0.340**	**0.180**	**0.200**	**0.270**	**0.190**
	Tandem repeats	rDNA	1.670	1.630	1.550	2.080	0.850	2.620	0.450	0.570	2.380	3.690	2.270	4.580
		Satellite	0.290	0.280	0.320	0.490	0.260	0.300	0.310	0.300	0.230	0.320	0.270	0.320
		**Total**	**1.960**	**1.910**	**1.870**	**2.570**	**1.110**	**2.920**	**0.760**	**0.870**	**2.610**	**4.010**	**2.540**	**4.900**
Annotated repetitive total			**12.420**	**11.760**	**12.050**	**43.560**	**46.110**	**46.380**	**38.230**	**43.960**	**46.480**	**44.050**	**43.810**	**48.110**
Unclassified_repeat			4.350	4.340	4.420	3.290	8.350	5.830	3.890	5.250	6.050	5.860	3.510	5.680
**Total**			16.770	16.10	16.47	46.85	54.460	52.210	42.120	49.210	52.530	49.910	47.320	53.790

Repeats		Lineage/class	Sp-OL-F			Sp-TE-M			Sp-TE-F	
			PI527332-F (%)	PI664498-F (%)	PI478393-F (%)	PI647861-M (%)	PI677114-M (%)	PI608712-M (%)	PI647861-F (%)	PI608712-F (%)
LTR retroelements	Ty1/Copia	Ale	0.240	0.190	0.280	0.220	0.180	0.160	0.275	0.210				
		Angela	13.060	17.380	13.880	10.260	21.040	19.870	12.770	16.770				
		Bianca	0.000	0.000	0.000	0.000	0.000	0.030	0.000	0.000				
		SIRE	0.190	0.460	0.260	0.070	0.700	0.770	0.110	0.370				
		TAR	0.070	0.260	0.200	0.020	0.310	0.320	0.065	0.270				
		Tork	0.000	0.010	0.010	0.000	0.010	0.030	0.000	0.000				
		Others	0.000	0.420	0.000	0.000	0.010	0.000	0.195	0.000				
		**Total**	**13.560**	**18.720**	**14.630**	**10.570**	**22.250**	**21.180**	**13.415**	**17.620**				
	Ty3/Gypsy	Athila	1.100	1.290	1.130	0.960	1.140	1.640	1.195	1.230				
		Ogre	16.770	15.310	16.540	15.420	18.380	17.040	17.125	16.060				
		Retand	0.000	5.540	6.420	0.000	4.630	4.100	6.425	6.230				
		CRM	0.180	0.290	0.200	0.060	0.360	0.360	0.180	0.230				
		Galadriel	0.130	0.140	0.190	0.110	0.030	0.000	0.130	0.100				
		Tekay	1.070	1.700	1.070	0.570	1.990	2.050	1.020	1.460				
		**Total**	**19.250**	**24.270**	**25.550**	**17.120**	**26.530**	**25.190**	**26.075**	**25.310**				
Other	LINE		0.050	0.080	0.040	0.000	0.090	0.080	0.000	0.050				
	Helitron		0.000	0.000	0.000	0.000	0.000	0.000	0.000	0.000				
	DNA transposons	EnSpm_CACTA	0.080	0.300	0.110	0.000	0.270	0.260	0.065	0.150				
		MuDR_Mutator	0.010	0.000	0.000	0.000	0.030	0.020	0.000	0.020				
		Tc1_Mariner	0.000	0.020	0.000	0.000	0.040	0.020	0.000	0.020				
		**Total**	**0.090**	**0.320**	**0.110**	0.000	**0.340**	**0.300**	**0.065**	**0.190**				
	Tandem repeats	rDNA	4.370	0.110	6.820	7.580	1.910	0.570	5.180	0.160				
		Satellite	0.350	0.280	0.250	0.360	0.230	0.290	0.360	0.230				
		**Total**	**4.720**	**0.390**	**7.070**	**7.940**	**2.140**	**0.860**	**5.540**	**0.390**				
Annotated repetitive total			**37.670**	**43.780**	**47.400**	**35.630**	**51.350**	**47.610**	**45.095**	**43.560**				
Unclassified_repeat			7.580	7.980	7.580	13.20	3.710	5.930	8.505	11.510				
**Total**			45.250	51.760	54.980	48.830	55.060	53.540	53.600	55.070				

The bold value indicates the total value of “Ty1/Copia”, “Ty3/Gypsy”, “DNA transposons”, “Tandem repeats” and “Annotated repetitive total”, respectively.

**FIGURE 2:**
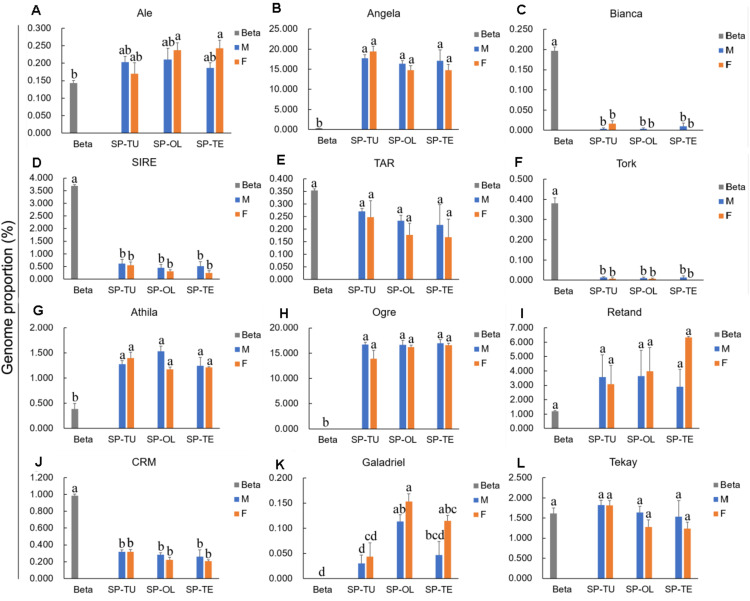
Comparison of *Ty1/Copia* and *Ty3/Gypsy* lineages proportion between spinach and sugar beet genome. **(A–F)** The proportion of *Ty1/Copia* repeats Ale, Angela, Bianca, SIRE, TAR and Tork in spinach and sugar beet genome. **(G–L)** The proportion of *Ty3/Gypsy* repeats Athila, Ogre, Retand, CRM, Galadriel and Tekay in spinach and sugar beet genome. “a, b, c, d,” means significance level by multiple comparison, *p* < 0.05.

For the *Ty1/Copia* repeats, the proportion of Angela was significantly higher in spinach than that in sugar beet, whereas the proportion of Bianca, SIRE, and TORK was significantly lower in spinach than that in sugar beet (Figure 2). For *Ty3/Gypsy* repeats, the proportion of Athila and Ogre are pronounced, while that in CRM was lower in spinach than that in sugar beet (Figure 2). Accordingly, Angela, Athila, and Ogre may contribute to the increasing proportion of repeats in the spinach genome.

### Repeat Proportion in *S. oleracea*, *S. turkestanica*, and *S. tetrandra* Genome

**FIGURE 3:**
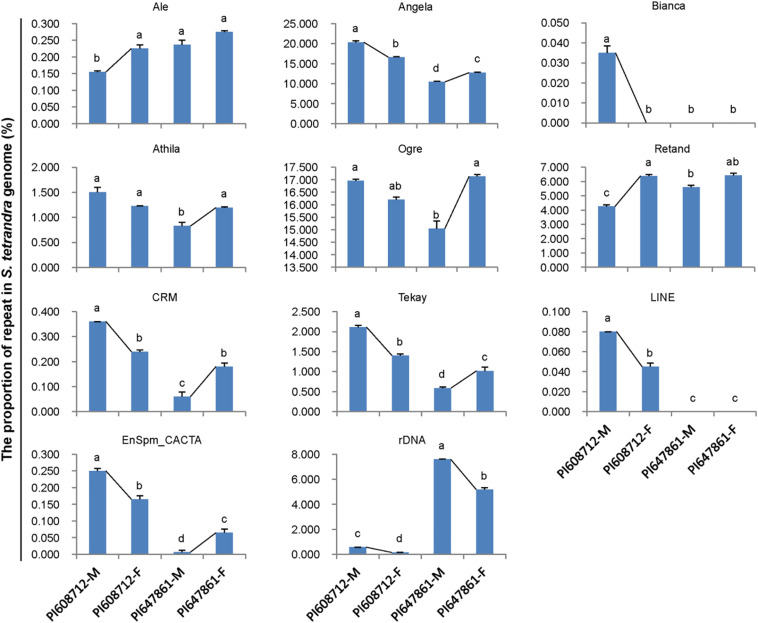
The differentially abundant repeats between male and female genome in two accessions of *S. tetrandra*. PI608712 with homomorphic sex chromosomes, PI647861 with heteromorphic sex chromosomes; PI608712-F, female genome of PI608712; PI608712-M, male genome of PI608712; PI647861-F, female genome of PI647861; PI647861-M, male genome of PI647861; the black line linking two bars indicates the variation trend; “a, b, c, d,” means significance level by multiple comparison, *p* < 0.05.

**FIGURE 4:**
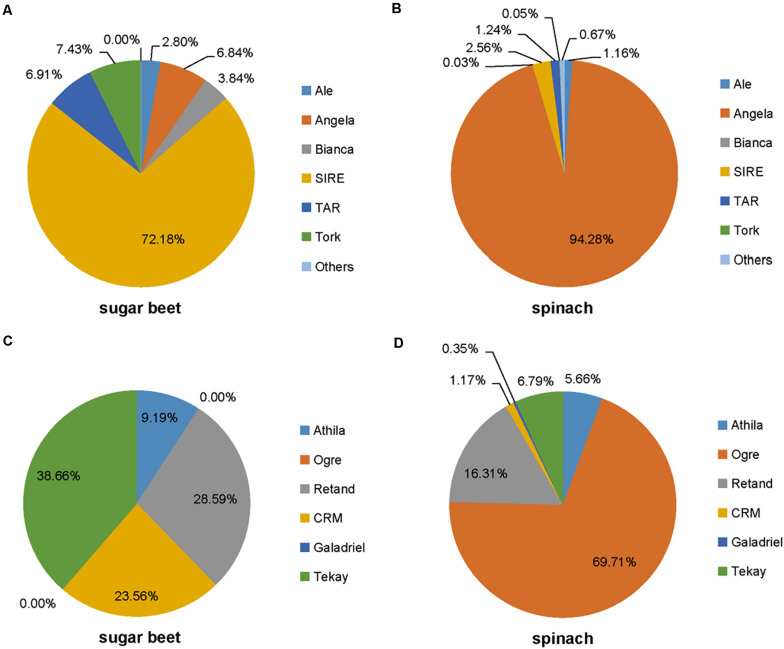
The abundance of each lineage in *Ty1/Copia* class **(A,B)** and *Ty3/Gypsy* class **(C,D)** in the spinach and sugar beet genomes.

Repetitive sequences are closely related to sex chromosome evolution. In the present, one *S. tetrandra* sample (PI647861) has heteromorphic sex chromosomes, while the sex chromosomes of the two other *S. tetrandra* samples and all *S. oleracea* and *S. turkestanica* samples are homomorphic. Assessment of the repeats proportions of genomes of *Spinacia* species revealed a conserved pattern between male and female genome of *S. oleracea* and S. *turkestanica* (Figure 2). However, in *S. tetrandra*, the proportion of several repeat lineages was significantly different between male and female genomes (Figure 3). For PI608712 with homomorphic sex chromosomes, the proportion of Ale and Retand was higher in the female genome than in the male genome, while the proportion of Angela, Bianca, CRM, Tekay, LINE, EnSpm_CACTA, and rDNA was higher in male genome than in the female genome (Figure 3). For PI647861 with heteromorphic sex chromosomes, the proportion of Angela, Athila, Ogre, CRM, Tekay, and EnSpm_CACTA was higher in the female genome than in the male genome, whereas the proportion of rDNA was higher in the male genome than in the female genome (Figure 4). Among these divergent repeat lineages, Angela, CRM, Tekay, EnSpm_CACTA, and rDNA are shared by PI608712 and PI647861, but an opposite trend of the shared repeats except rDNA is found between PI608712 and PI647861 (Figure 3). The proportion of rDNA is higher in male genome than in the female genome of PI608712 and PI647861. In addition, Ale, Retand, Bianca, and LINE are differentially abundant only in PI608712, while Athila and Ogre are differentially abundant only in PI647861.

### Comparative Analysis of Interspersed Repeats

**FIGURE 5:**
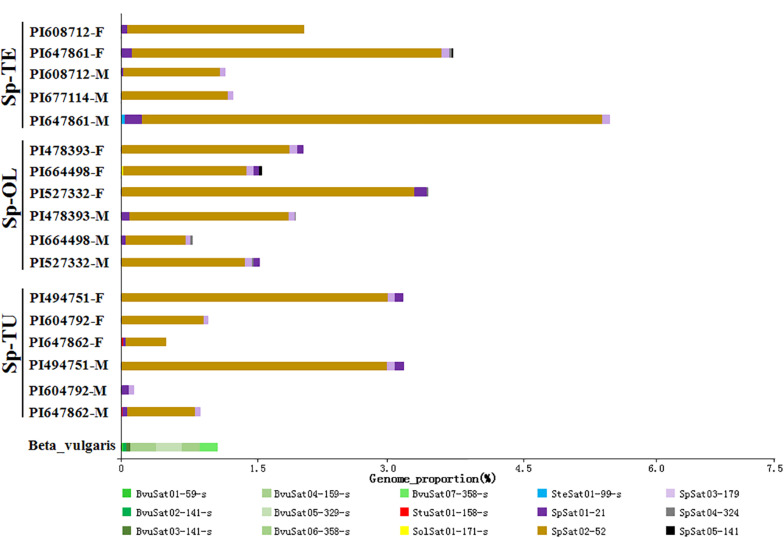
Estimated satellite abundance in spinach and sugar beet genome. The size of the rectangle is proportional to the number of reads in a cluster for each species. Colors of the rectangles correspond to repeat type. See Table 1 for species codes.

The repetitive element portions of genomes from sugar beet and spinach are mainly contributed by LTR retrotransposons (Table 1 and Figure 1B). In *Spinacia* species, Angela is the predominant lineage among *Ty1/Copia* elements, accounting for more than 94.28%; in *B. vulgaris*, SIRE lineage accounts for the majority (72.18%) of *Ty1/Copia* elements (Figure 5). For *Ty3/Gypsy* elements, Ogre is the most common type of lineages (69.71%) in *Spinacia* species; in *B. vulgaris*, Retand, and Tekay lineages form a relatively large proportion, accounting for 28.59 and 38.66%, respectively (Figure 4). Moreover, several LTR variants, namely, Ogre, Galadriel, LINE, and Tc1_Mariner exclusively appear in *Spinacia* species; however, Helitron only appears in *B. vulgaris* (Table 1 and Supplementary Table 4).

### Comparative Analysis of Major Groups of Satellite

The proportion of satellite repeats was found approximately 0.3% in both the spinach and sugar beet genomes (Table 1), and the satellites which detected by RepeatExplorer and TAndem REpeat ANalyzer (TAREAN) are summarized in Supplementary Table 5. A total of 15 satellite DNA families were found (Figure 5 and Supplementary Table 5). The monomer size of the satellite DNAs is broad and ranged from 21 to 358 bp, among the four species (Supplementary Table 6). Subsequent analyses revealed that BvuSat01-59-s (MN918718), BvuSat02-141-s (MN918719), BvuSat03-141-s (MN918720), BvuSat04-159-s (MN918721), BvuSat05-329-s (MN918722), BvuSat06-358-s (MN918723), and BvuSat07-358-s (MN918724) were only present in the *B. vulgaris* genome. Previously, many satellite DNAs have been characterized in the sugar beet genome. Herein, we blasted the satellite DNAs identified in this work against these in GenBank (Table 2). BvuSat01-59-s, BvuSat02-141-s, BvuSat03-141-s, and BvuSat06-358-s had no similarities with other known sequences (Table 2). However, BvuSat04-159-s, BvuSat05-329-s, and BvuSat07-358-s shared a higher similarity (94.94, 93.62, and 96.04%) with sugar beet satellite pEV1-like sequence (JN172936.1), pBV1-like sequence (JN172938.1), and pAv34-1 (AM076742.1), respectively (Table 2). Hence, among these seven satellite DNAs identified in the sugar beet genome, four were novel DNAs in current work. In *Spinacia* genomes, eight satellite DNAs were identified (Figure 5 and Table 2). Five satellite DNAs, SpSat01–21 (5’-GCTATCGGCACCCGCCAACTA-3’), SpSat02-52 (MN918725), SpSat03-179 (MN918726), SpSat04-324 (MN918727), and SpSat05-141 (MN918728), were found in three *Spinacia* species (Figure 5). However, StuSat01-158-s (MN918729) was only found in the *S. turkestanica* genome; SolSat01-171-s (MN918730) was unique in the *S. oleracea* genome; SteSat01-99-s (MN918731) was only found in the *S. tetrandra* genome. Among the common satellite DNAs in *Spinacia* species, SpSat02-52 is the most abundant DNA.

**TABLE 2:** Blast search for satellite DNAs.

Satellite DNA	BLASTn against Nt	*E*-value	Idendity	Annotation
BvuSat01-59-s	JN172936.1	1E–65	94.94%	Sugar beet satellite pEV1-like sequence
BvuSat02-141-s	None	−	−	−
BvuSat03-141-s	None	−	−	−
BvuSat04-159-s	None	−	−	−
BvuSat05-329-s	JN172938.1	6E–137	93.62%	Sugar beet satellite pBV1-like sequence
BvuSat06-358-s	None	−	−	−
BvuSat07-358-s	AM076742.1	9E–148	96.04%	Sugar beet satellite pAv34-1
SpSat01-21	CP025675.1	0.12	100.00%	*S. oleracea* proline-rich extensin-like protein EPR1
SpSat02-52	None	−	−	−
SpSat03-179	None	−	−	−
SpSat04-324	None	−	−	−
SpSat05-141	None	−	−	−
StuSat01-158-s	None	−	−	−
SolSat01-171-s	XR_002529869.1	2E–32	90.99%	*S. oleracea* uncharacterized LOC110775734
SteSat01-99-s	None	−	−	−

*Nt: Nucleotide collection in NCBI.*

### Chromosomal Localization of Satellite DNAs

**FIGURE 6:**
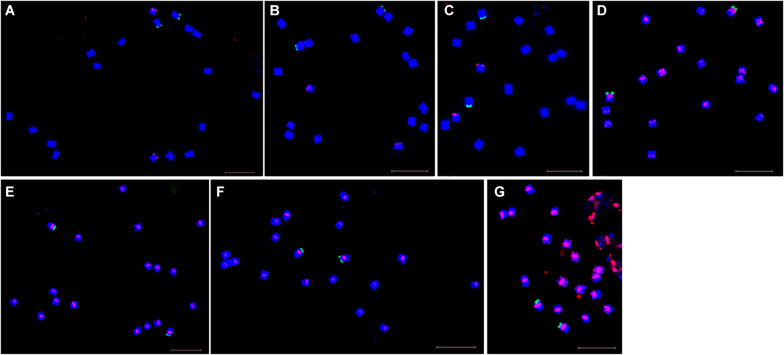
Localization of seven satellites on metaphase chromosomes of *B. vulgaris* using fluorescence *in situ* hybridization. Blue are DAPI stained chromosomes, green signals show chromosomal localization of 45 rDNA, The satellite DNAs were labeled with Texas red (red signal). **(A)** Probe BvuSat01-59-s (red). **(B)** Probe BvuSat02-141-s. **(C)** Probe BvuSat03-141-s. **(D)** Probe BvuSat04-159-s. **(E)** Probe BvuSat05-329-s. **(F)** Probe BvuSat06-358-s, **(G)** Probe BvuSat07-358-s. Bar indicates 10 μm.

**FIGURE 7:**
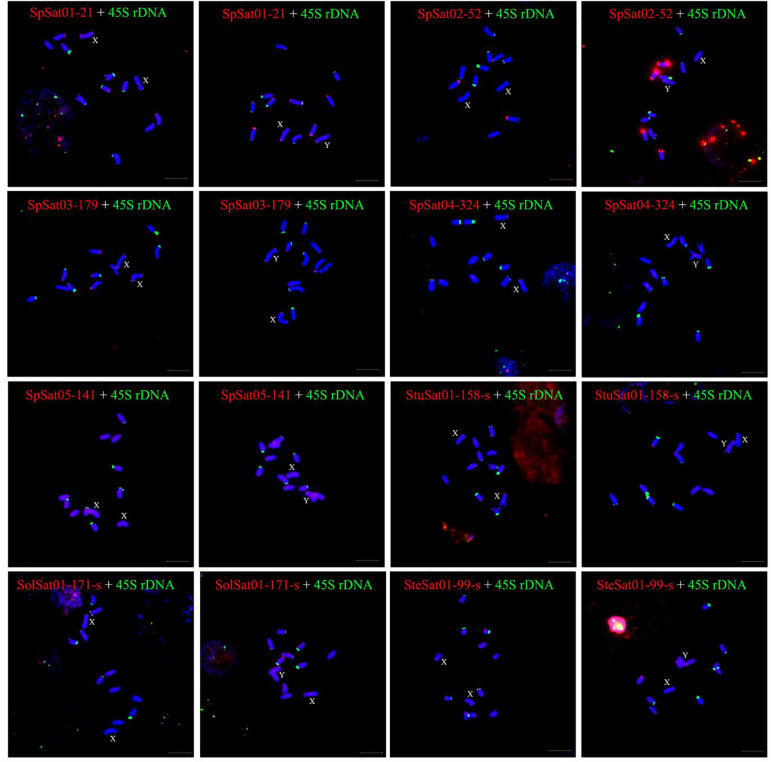
Localization of eight satellites on metaphase chromosomes of spinach using fluorescence *in situ* hybridization. The name of satellites and sex of individual are indicated inside each figure. Blue are DAPI stained chromosomes, the satellite DNAs were labeled with Texas red (red signal), and green signals show chromosomal localization of satellites. Bar indicates 10 μm.

**FIGURE 8:**
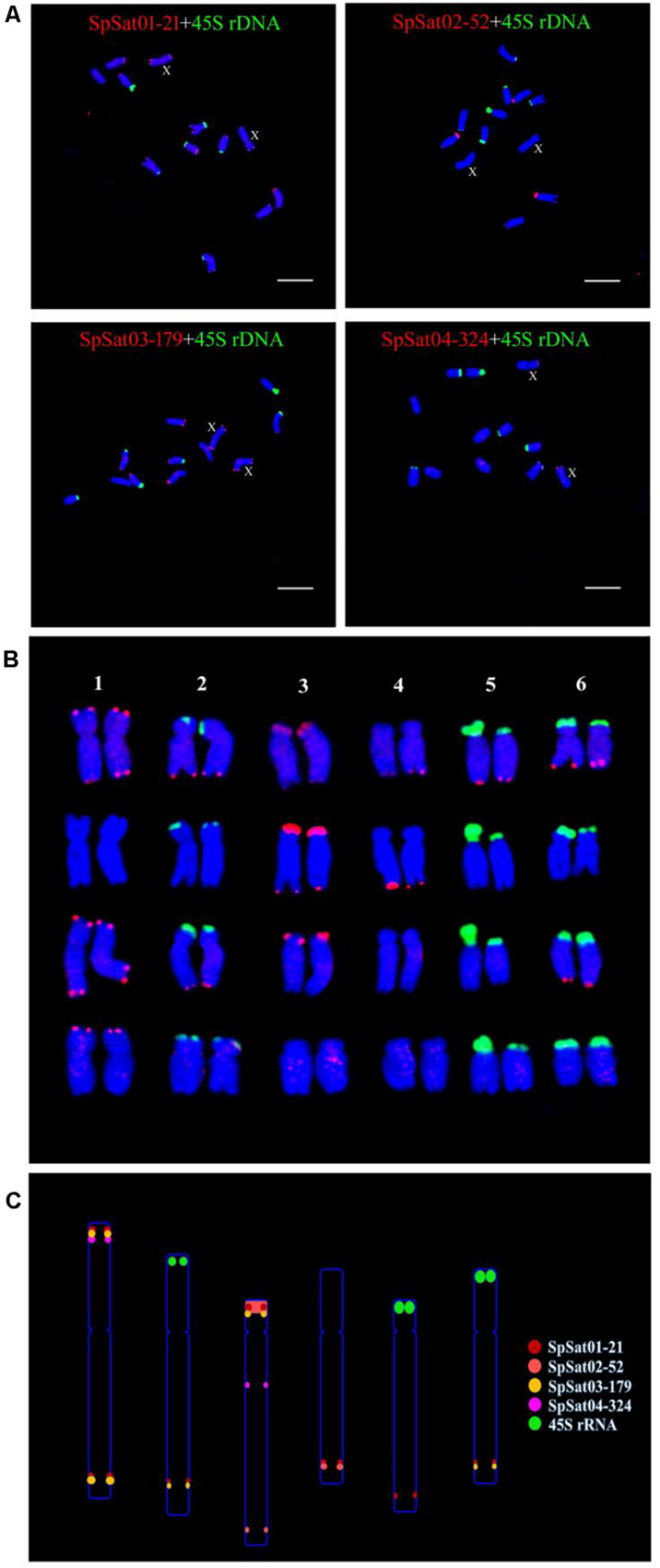
Karyotype and ideograph for spinach mitotic metaphase chromosomes. **(A)** FISH analysis of SpSat01-21, SpSat02-52, SpSat03-179, and SpSat04-324 (red), and 45S rDNA (green) on mitotic chromosomes. Arrows indicate the sex chromosomes. **(B)** Karyotype analysis of spinach based on the size and FISH signal pattern. **(C)** Ideogram shows the positions of SpSat01-21 (red), SpSat02-52 (yellow), SpSat03-179 (pink), SpSat04-324 (purple), and 45S rDNA (green). Bars = 10 μm.

Fluorescence *in situ* hybridization (FISH) was used to analyze the chromosomal localization of the 15 satellites identified above in *B. vulgaris*, male *S. oleracea* and female *S. oleracea* mitotic metaphase chromosomes (Figures 6, 7). The hybridization revealed that BvuSat01-59-s, BvuSat02-141-s, and BvuSat03-141-s, are localized to the telomeric regions of a pair of chromosome (Figures 6A–C); BvuSat04-159-s is located in the intercalary positions of each chromosome-arm (Figure 6D); BvuSat05-329-s and BvuSat06-358-s are associated with the centromere (Figures 6E,F), and BvuSat07-358-s occurs on most chromosome ends with signals on the centromere of each chromosome (Figure 6G). However, all seven satellite DNA sequences identified in sugar beet did not show fluorescence signal when hybridized with the spinach mitotic metaphase chromosomes. For the eight spinach satellite DNA sequences (Figure 7), SpSat01-21 was localized to the telomeric regions of the sex chromosome (Chr 1) and the long arm of Chr 2, 3, 4, 5, and 6; SpSat02-52 was mostly concentrated in the telomeric regions of the Chr 3 and the long arm of Chr 4; hybridization signal of SpSat03-179 was mostly enriched on the telomeric regions of the sex chromosome (Chr 1) and the long arm of Chr 2, 3, and 6; SpSat04-324 and SpSat05-141 are featured by the distribution of hybridization signals on the telomeric region of the sex chromosome (Chr 1) short arm; the signals of StuSat01-158-s and SolSat01-171-s were found on the telomeric regions of the sex chromosome (Chr 1) and the long arm of Chr 2, 5, and 6; and the signals of SteSat01-99-s concentrated on the long arm of Chr 2 and 5. Signals of these eight satellites have notable difference between male and female *S. oleracea* mitotic metaphase. No fluorescence signals were found from hybridizations between eight satellite DNA sequences and *B. vulgaris* mitotic metaphase chromosomes, indicating that these repeats are *Spinacia* species specific. Satellite DNA sequences (SpSat01-21, SpSat02-52, SpSat03-179, SpSat04-324, and 45S rDNA) were used as probes, and sequential FISH was used to establish accurate spinach karyotype (Figure 8A). These four satellite clusters featured a star-like or circular graph topology (Supplementary Figures 1, 2). As shown in Figures 8B,C, Chr 1 pair: SpSat01-21 and SpSat03-179 on the telomeric region of both arms and SpSat04-324 on the telomeric region of short arm; Chr 2 pair: SpSat01-21 and SpSat03-179 on the telomeric region of long arm and 45S rDNA on the telomeric region of short arm; Chr 3 pair: SpSat02-52 on the telomeric region of both arms, SpSat01-21 and SpSat03-179 on the telomeric region of short arm; Chr 4 pair: SpSat01-21 and SpSat02-52 on the telomeric region of long arm; Chr 5 pair: SpSat01-21 on the telomeric region of long arm and 45S rDNA on the telomeric regions of short arm; Chr 6 pair: SpSat01-21 and SpSat03-179 on the telomeric region of long arm and 45S rDNA on the telomeric region of short arm. Thus, distribution characters of eight satellite DNAs are different among each chromosome pair, which can be used as DNA markers for accurate karyotype of spinach.

## Discussion

### Genome Size Variation and Repeat Content

A recent sequenced draft sugar beet reference genome comprised 567 Mb, 42.3% of which were identified as repetitive sequences (Dohm et al., 2014). The spinach draft genome size is approximately 996 Mb, with 74.4% repetitive sequences (Xu et al., 2017). However, *De novo* sequencing only identified 16.45% of the total sugar beet genome (Organelle genome excluded) as repetitive DNA sequences; in *Spinacia* species genome, the repetitive sequences proportion is 49.56, 50.50, and 53.41%, respectively. The repeats proportion in our results is lower than that reported in previous studies, which might be attributed to the different methods for repeats annotation. Nevertheless, our results were consistent with Kudoh group’s report, which revealed a significantly higher proportion of repeats in *Spinacia* species compared with *B. vulgaris* (Kudoh et al., 2018). Moreover, the proportion of the LTR retrotransposon *Ty3/Gypsy* and *Ty1/Copia* was obviously higher in spinach genome than that in sugar beet genome (Figure 1B). This result is consistent with that LTR-retrotransposons may be related to genome size variation between the close plant relatives (Bennetzen et al., 2005; Kowar et al., 2016).

### Sex Chromosome Evolution and Repeat Elements

In *Spinacia*, sex chromosomes among three of the *S. tetrandra* accessions are heteromorphic but the other *Spinacia* species are homomorphic s; thus, spinach provides a good opportunity for studying transition of homomorphic sex chromosomes to heteromorphic sex chromosomes The abundance of repetitive sequence around sex-determining loci can suppress the homologous recombination of sex-determining loci between X and Y chromosomes, and finally form the male-specific region (Li et al., 2016). Hence, variations of repeats proportions between female and male genomes can reflect differences between X and Y chromosomes. To understand the roles of repetitive DNA in spinach sex chromosome evolution, we characterized the differentially accumulated repeats elements in male and female spinach. Comparison of the repeats proportion between female and male genomes of three *Spinacia* species indicated that the difference was present in *S. tetrandra* samples but not in the *S. oleracea* or *S. turkestanica* samples. Herein, we analyzed two accessions of *S. tetrandra*, PI608712 with homomorphic sex chromosomes and PI647861 with heteromorphic sex chromosomes. *S. oleracea* and *S. turkestanica* samples with homomorphic sex chromosomes are at the early stage of sex chromosome evolution, which may result negligible differences of repeat proportion between female and male genome. However, in *S. tetrandra*, several repeats (Ale, Retand, Angela, Bianca, CRM, Tekay, LINE, EnSpm_CACTA, and rDNA) exhibit different abundances between the female and male genomes of PI647861 although its sex chromosomes are homomorphic (Figure 3). For PI647861, Angela, Athila, Ogre, CRM, Tekay, EnSpm_CACTA, and rDNA are differentially abundant repeats between female and male genomes (Figure 3). Among these differentially abundant repeats, only tandem repeat rDNA showed a higher proportion in male genome both of PI608712 and PI647861. We found that the proportions of Ale, Bianca, Athila, Ogre, Retand, and CRM were significantly different in the male genome but not in the female genome between PI608712 and PI647861 (Figure 4); compared with the male genome of PI608712, only the proportion of Ale and Retand was increased in the male genome of PI647861. We presumed that repetitive DNA sequences are correlated with the formation of sex chromosomes and the transition from homomorphic sex chromosomes to heteromorphic sex chromosomes in *Spinacia* species. The sex determination region (SDR) of spinach was identified (Yu et al., 2020). Meanwhile, k-mer analysis was useful in identifying the sex determination region in persimmons (Akagi et al., 2014) and date palm (Torres et al., 2018) efficiently. In the future, we will use this method to clone the SDR of male and female species of *Spinacia* and explore the function of repetitive DNA sequences on sex chromosome evolution in spinach.

### Interspersed Repeats

The LTR retroelements, particularly the *Ty3/Gypsy* class, are the major contributions to the repetitive sequences in spinach genome (Xu et al., 2017). Based on the sequence composition of the largest 18 superclusters, superclusters 8 (Ogre), 11 (Galadriel), 13 (LINE), and 17 (MuDR_Mutator) only appear in *Spinacia* species and not in sugar beet. Thus, we proposed that insertion frequency of these elements, especially Ogre (abundant in female and specific to spinach), may be correlated with the mating system. *Spinacia* species are commonly dioecious and their offspring are obtained by outcrossing among different sexes. In *B. vulgaris*, diploid species are hermaphroditic. The single cluster Helitron was only found in *B. vulgaris* and not in *Spinacia* species. Helitron, a rolling-circle DNA transposon, plays an important role in plant evolution. As reported in *Brassica napus*, function loss of *BnSP11-1*, the self-incompatibility male determining gene, is due to the insertion of a 3.6 kb non-autonomous Helitron transposon into this gene promoter (Gao et al., 2016). Hence, further experiments will be needed to study the role of Ogre and Helitron transposon in spinach sex chromosome evolution.

### Satellite Repeats and Sex Chromosome Evolution of Spinach

Satellite accumulation accompanies sex chromosomes evolution in some plant species with heteromorphic sex chromosomes. Compared with mammalian sex chromosomes that evolved 300 Mya, sex chromosomes in plants are incipient which ranges from 3 to 13 Mya (Navajas-Perez et al., 2005; Sousa et al., 2013; Kubat et al., 2014). In *S. latifolia*, TRAYC and STAR-Y satellites accumulated on the Y chromosome (Hobza et al., 2006, 2007). In *Rumex acetosa*, RA160 and RA690 satellites are enriched X and Y chromosomes, respectively (Steflova et al., 2013). Seabuckthorn (*Hippophae rhamnoides*) has a large X chromosome and a small Y chromosome; HRTR8 satellite is specific to the X chromosome, while HRTR12 satellite is Y-specific (Puterova et al., 2017). Satellites accumulate not only in the heterochromatic regions of the sex chromosomes but also in the euchromatic region. A model has revealed the accelerated spread of TEs by microsatellite targeting. However, the rate of satellites spreading during sex chromosome evolution and their roles in heterochromatinization process remain unclear. In *Spinacia*, the genome size and karyotype of *S. tetrandra* are clearly distinct from those of two *Spinacia* members. The sex chromosome pairs of three *S. tetrandra* accessions are heteromorphic, while sex chromosomes of the other *Spinacia* members are homomorphic; In the present study, the signals of six satellites (SpSat01-21, SpSat03-179, SpSat04-324, SpSat05-141, StuSat01-158-s, and SolSat01-171-s) were located on chromosome 1 (Figure 7). Our previous study showed that two satellites, namely, Spsat2 (has similar hybridization signal but different sequences with SpSat01-21) and Spsat3 provide hybridization signals on chromosome 1 (Li et al., 2019). Chromosome 1 is a sex chromosome of spinach (Deng et al., 2012, 2013; Okazaki et al., 2019). The SDR, which is approximately 18.4 Mb, is located on the short arm of Chromosome 1 (Yu et al., 2020), as consistent as signals of six satellites we developed in current study (SpSat01-21, SpSat03-179, SpSat04-324, SpSat05-141, StuSat01-158-s, and SolSat01-171-s) (Figure 7). Searching DNA sequences of the sex satellites against the DNA sequences of SDR achieved 141 copies of SolSat01-171-s in SDR, and the standard cutoff is Percentage of identical matches ≥ 90% and *E*-value ≤ 3.61E^–61^ (Supplementary Table 7). Thus, we speculated that the accumulation of satellite DNA on the short arm of chromosome 1 may be involved in the sex chromosome evolution in *Spinacia* species. However, the signals of the eight satellites between male and female *S. oleracea* mitotic metaphase have notable differences (Figure 7). This result may be related to the early stages of sex chromosome evolution in spinach. The sex chromosomes were accompanied by insufficient recombination suppression induced by repetitive sequence insertions. In our future work, we will use the eight satellite DNA sequences to hybridize with mitotic metaphase chromosomes of *S. turkestanica*, *S. oleracea*, and *S. tetrandra* to study the role of satellites in the sex chromosome evolution.

In conclusion, the global distribution of the major repetitive sequence landscape in the male and female genome of *Spinacia* species was characterized using next-generation sequencing data. Compared with that of sugar beet, the genome of spinach showed an increased total proportion, which is largely attributed to the accumulation of LTR retrotransposon *Ty3/Gypsy* (especially Athila and Ogre) and *Ty1/Copia* (especially Angela). Compared with *B. vulgaris*, *Spinacia* species had a higher percentage of repeat elements potentially derived from a recent lineage-specific repeat burst. Among *Ty1/Copia* retro-transposable elements, Angela lineage was predominant, accounting for more than 94.97% of the *Ty1/Copia* elements in *Spinacia* species; SIRE lineage was more popular (> 71.44%) among *Ty1/Copia* elements in sugar beet. Distribution of *Ty3/Gypsy* retro-transposable elements showed that Ogre was the most common type (69.28%) in *Spinacia* species; Retand and Tekay lineages are the main types among *Ty3/Gypsy* elements in sugar beet, accounting for 28.5 and 38.6%, respectively. Development of seven sugar beet-specific and eight spinach-specific satellite DNA probes provided evidence of repetitive sequence divergence between two genera; furthermore, the six satellite DNA probe signals from *Spinacia* species were identified on the short arm of the sex chromosome by FISH. A total of 141 copies of SolSat01-171-s were present in SDR. Thus, we proposed that the accumulation of satellite DNA on the short arm of chromosome 1 may be involved in the sex chromosome evolution of *Spinacia* species.

## Data Availability Statement

The datasets presented in this study can be found in online repositories. The names of the repository/repositories and accession number(s) can be found below: https://www.ncbi.nlm.nih.gov/bioproject/PRJNA608209/.

## Author Contributions

NL, XL, and CD designed the experiments. NL, XL, JZ, and CD conducted the study, processed the data, and wrote the manuscript. LY, SL, YZ, RQ, WG, and CD discussed the results and revised the manuscript. All authors have read and approved the final manuscript.

## Conflict of Interest

The authors declare that the research was conducted in the absence of any commercial or financial relationships that could be construed as a potential conflict of interest.

## Footnotes

1 https://npgsweb.ars-grin.gov/gringlobal/query/query.aspx

2 https://repeatexplorer-elixir.cerit-sc.cz/galaxy/

3 https://www.ncbi.nlm.nih.gov/sra/?term=SRR952972

## Abbreviations

NGS: Next-Generation Sequencing
FISH: fluorescence *in situ* hybridization
LTR: Long terminal repeat
TAREAN: TAndem REpeat Analyzer
SDR: Sex determination region.
