# Supplementary material for: Genome-Wide Analysis of Transposable Elements and Satellite DNAs in Spinacia Species to Shed Light on Their Roles in Sex Chromosome Evolution
Source: Front Plant Sci. 2021 Jan 14;11:575462. doi: 10.3389/fpls.2020.575462 (PMC7840529; doi:10.3389/fpls.2020.575462)

Supplementary Materials

Supplementary Table 1 The details of plant specimens.

Species	Plant ID	Plant Name	Origin	Identifier
S. oleracea L.	PI 527332	DA JIAN YE BO CAI	China	Vegetable and Floral Research Institute, Chinese Academy of Agricultural Science
	PI 664498	Cornell ID #273	China	Tong Daxiang, Institute of Crop Germplasm Resources, Chinese Academy of Agricultural Sciences
	PI 478393	Cornell ID #174	China	T. J. Orton, Department of Vegetable Crops University of California
S. turkestanica Ilj.	PI 647862	1.1	Ahal, Turkmenistan	Muhamet Durikov and K. Mamedov, Ministry of Nature Protection of Turkmenistan, National Institute of Deserts, Flora, and Fauna
	PI 604792	SPI 155/83	Germany	Specht, Carl-Eckhard Institut fur Pflanzengenetik und Kulturpflanzenforschung
	PI 494751	VIR 490	Uzbekistan	N.I. Vavilov Institute of Plant Industry
S. tetrandra Stev.	PI 647861	1.4	Georgia	Maia Akhalkatsi and Marine Mosulishvili, Institute of Botany, Georgian Academy of Sciences
	PI 677114	1.1	Georgia	Maia Akhalkatsi and Marine Mosulishvili, Institute of Botany, Georgian Academy of Sciences
	PI 608712	Cornell ID #148	Germany	Karl Hammer, Inst. fur Pflanzengenetik und Kulturpflanzenforschung

Supplementary Table 2 Statistics of whole-genome shotgun sequencing reads of *Spinacia* species.

Sample_ID	Raw_Reads	Raw_Bases	Valid_Reads	Valid_Bases	Valid%	Q20%	Q30%	GC%
Sp-OL-F-1	170817344	25.62G	164240284	24.60G	96.00	96.76	92.09	46.70
Sp-OL-F-2	150379056	22.56G	146911292	22.02G	97.62	97.17	92.85	44.40
Sp-OL-F-3	232622452	34.89G	223948664	33.56G	96.17	96.54	91.55	46.91
Sp-OL-M-1	161377266	24.21G	156371252	23.44G	96.82	96.78	92.02	44.35
Sp-OL-M-2	176670378	26.50G	173818170	26.06G	98.33	97.40	93.32	41.71
Sp-OL-M-3	176736794	26.51G	171063554	25.63G	96.69	96.78	92.02	46.29
Sp-TE-F-1	167118144	25.07G	161127806	24.13G	96.27	96.77	92.08	46.96
Sp-TE-F-3	134145844	20.12G	128047162	19.19G	96.36	96.78	92.09	45.68
Sp-TE-M-1	139216170	20.88G	131798018	19.73G	94.46	96.08	90.72	48.61
Sp-TE-M-2	249574760	37.44G	243923478	36.56G	97.67	97.13	92.74	43.07
Sp-TE-M-3	138224738	20.74G	133630780	20.03G	97.38	97.14	92.81	42.65
Sp-TU-F-1	204627098	30.69G	201247882	30.17G	98.31	97.35	93.27	39.39
Sp-TU-F-2	135365482	20.30G	132273814	19.83G	97.66	97.07	92.65	41.97
Sp-TU-F-3	146784154	22.02G	142381348	21.33G	97.17	97.00	92.58	45.46
Sp-TU-M-1	206706180	31.01G	202412512	30.35G	97.88	97.07	92.63	41.70
Sp-TU-M-2	140804130	21.12G	136553422	20.46G	96.66	96.67	91.82	43.95
Sp-TU-M-3	177388058	26.61G	171497954	25.69G	96.54	96.92	92.40	45.59

Supplementary Table 3 RepeatExplorer analyses of *B. vulgaris* and three *Spinacia* species.

						Beta_vulgaris_1		Beta_vulgaris_9		Beta_vulgaris	
						Genome_proportion[%]	Nreads	Genome_proportion[%]	Nreads	Genome_proportion[%]	Nreads
Unclassified_repeat						4.350	20759.000	4.340	20523.000	4.420	21533.000
rDNA					45S_rDNA	1.270	6069.000	1.240	5889.000	1.180	5771.000
					25S_rDNA	0.000	0.000	0.000	0.000	0.000	0.000
					5S_rDNA	0.400	1915.000	0.390	1846.000	0.370	1790.000
					rDNA	1.670	7984.000	1.630	7735.000	1.550	7561.000
satellite						0.290	1402.000	0.280	1324.000	0.320	1582.000
mobile_element	Class_I	LTR			Ty1_copia	0.000	0.000	0.000	0.000	0.000	0.000
			Ty1_copia		Ale	0.160	753.000	0.140	649.000	0.130	625.000
					Angela	0.360	1738.000	0.340	1606.000	0.350	1719.000
					Bianca	0.180	860.000	0.220	1043.000	0.190	906.000
					SIRE	3.740	17826.000	3.580	16926.000	3.760	18313.000
					TAR	0.350	1690.000	0.340	1599.000	0.370	1817.000
					Tork	0.440	2117.000	0.320	1500.000	0.380	1869.000
					Total Ty1_copia	5.230	24984.000	4.940	23323.000	5.180	25249.000
				Ty3_gypsy	Athila	0.650	3114.000	0.220	1062.000	0.280	1376.000
					Ogre	0.000	0.000	0.000	0.000	0.000	0.000
					Retand	1.310	6242.000	1.110	5230.000	1.160	5663.000
					CRM	0.980	4666.000	0.940	4464.000	1.030	5011.000
					Galadriel	0.000	0.000	0.000	0.000	0.000	0.000
					Tekay	1.280	6102.000	1.850	8748.000	1.710	8326.000
					Total Ty3_gypsy	4.220	20124.000	4.120	19504.000	4.180	20376.000
					LINE	0.000	0.000	0.000	0.000	0.000	0.000
	Class_II	Subclass_1	TIR		EnSpm_CACTA	0.810	3883.000	0.760	3574.000	0.800	3918.000
					MuDR_Mutator	0.150	720.000	0.010	50.000	0.000	0.000
			Tc1_Mariner			0.000	0.000	0.000	0.000	0.000	0.000
		Subclass_2			Helitron	0.050	216.000	0.020	73.000	0.020	102.000
					Total	16.770	80072.000	16.100	76106.000	16.470	80321.000

SP-TU-M-1-3		SP-TU-M-2-1		SP-TU-M-3-1		SP-TU-F-1-1		SP-TU-F-2-3		SP-TU-F-3-1	
Genome_proportion[%]	Nreads	Genome_proportion[%]	Nreads	Genome_proportion[%]	Nreads	Genome_proportion[%]	Nreads	Genome_proportion[%]	Nreads	Genome_proportion[%]	Nreads
3.290	11009.000	8.350	25233.000	5.830	14281.000	3.890	15904.000	5.250	17604.000	6.050	15217.000
0.470	1556.000	0.720	2170.000	0.570	1390.000	0.400	1632.000	0.470	1578.000	0.510	1282.000
1.540	5160.000	0.000	0.000	1.870	4591.000	0.000	0.000	0.000	0.000	1.740	4374.000
0.070	229.000	0.130	400.000	0.180	433.000	0.050	201.000	0.100	336.000	0.130	323.000
2.080	6945.000	0.850	2570.000	2.620	6414.000	0.450	1833.000	0.570	1914.000	2.380	5979.000
0.490	1641.000	0.260	795.000	0.300	743.000	0.310	1272.000	0.300	991.000	0.230	579.000
0.250	840.000	0.430	1304.000	0.060	158.000	0.000	0.000	0.000	0.000	0.350	889.000
0.170	560.000	0.200	618.000	0.240	579.000	0.110	439.000	0.160	546.000	0.240	603.000
19.340	64660.000	18.020	54474.000	15.880	38895.000	21.600	88396.000	20.220	67829.000	16.520	41565.000
0.000	0.000	0.010	30.000	0.000	0.000	0.020	93.000	0.030	111.000	0.000	0.000
0.930	3119.000	0.660	1983.000	0.250	610.000	0.800	3273.000	0.610	2040.000	0.230	577.000
0.290	985.000	0.280	861.000	0.240	596.000	0.290	1206.000	0.360	1206.000	0.090	216.000
0.010	37.000	0.020	66.000	0.010	24.000	0.000	0.000	0.000	0.000	0.020	47.000
20.740	69361.000	19.190	58032.000	16.620	40704.000	22.820	93407.000	21.380	71732.000	17.100	43008.000
1.440	4804.000	1.230	3721.000	1.160	2840.000	1.690	6923.000	1.220	4079.000	1.280	3211.000
15.630	52237.000	17.270	52203.000	17.190	42097.000	10.550	43157.000	13.740	46088.000	17.510	44043.000
6.170	19451.000	4.280	12939.000	6.440	15760.000	0.000	0.000	3.820	12805.000	5.410	13599.000
0.360	1194.000	0.330	986.000	0.260	629.000	0.300	1221.000	0.380	1267.000	0.270	675.000
0.070	236.000	0.020	71.000	0.000	0.000	0.020	62.000	0.000	0.000	0.110	289.000
2.020	6757.000	1.920	5819.000	1.530	3757.000	1.840	7515.000	2.050	6875.000	1.560	3933.000
25.690	84679.000	25.050	75739.000	26.580	65083.000	14.400	58878.000	21.210	71114.000	26.140	65750.000
0.120	400.000	0.070	215.000	0.000	0.000	0.050	219.000	0.160	521.000	0.100	243.000
0.250	836.000	0.220	654.000	0.180	452.000	0.160	645.000	0.270	893.000	0.180	448.000
0.050	175.000	0.010	33.000	0.000	0.000	0.010	40.000	0.030	117.000	0.000	0.000
0.050	174.000	0.030	97.000	0.020	38.000	0.030	104.000	0.040	122.000	0.000	0.000
0.010	35.000	0.000	0.000	0.000	0.000	0.000	0.000	0.000	0.000	0.000	0.000
55.720	175642.000	54.460	164672.000	52.210	127873.000	53.000	163564.000	49.210	165008.000	52.530	132113.000

SP-OL-M-1-4-1		SP-OL-M-2-1		SP-OL-M-3-1		SP-OL-F-1-2-3		SP-OL-F-2-3		SP-OL-F-3-1	
Genome_proportion[%]	Nreads	Genome_proportion[%]	Nreads	Genome_proportion[%]	Nreads	Genome_proportion[%]	Nreads	Genome_proportion[%]	Nreads	Genome_proportion[%]	Nreads
5.860	17883.000	3.510	12558.000	5.680	16055.000	7.580	17472.000	7.980	24719.000	7.580	21404.000
0.740	2256.000	0.440	1587.000	0.000	0.000	0.910	2093.000	0.000	0.000	0.000	0.000
2.790	8519.000	1.770	6349.000	4.380	12370.000	3.270	7533.000	0.000	0.000	6.610	18663.000
0.160	481.000	0.060	203.000	0.200	554.000	0.190	441.000	0.110	330.000	0.210	591.000
3.690	11256.000	2.270	8139.000	4.580	12924.000	4.370	10067.000	0.110	330.000	6.820	19254.000
0.320	975.000	0.270	952.000	0.320	906.000	0.350	795.000	0.280	855.000	0.250	709.000
0.030	97.000	0.000	0.000	0.280	794.000	0.000	0.000	0.420	1307.000	0.000	0.000
0.210	648.000	0.140	498.000	0.280	790.000	0.240	558.000	0.190	589.000	0.280	801.000
16.780	51226.000	17.790	63708.000	14.430	40757.000	13.060	30081.000	17.380	53859.000	13.880	39214.000
0.000	0.000	0.010	45.000	0.000	0.000	0.000	0.000	0.000	0.000	0.000	0.000
0.390	1205.000	0.750	2675.000	0.220	617.000	0.190	449.000	0.460	1421.000	0.260	733.000
0.190	572.000	0.280	1020.000	0.230	637.000	0.070	166.000	0.260	792.000	0.200	562.000
0.000	0.000	0.020	83.000	0.010	37.000	0.000	0.000	0.010	30.000	0.010	34.000
17.570	53651.000	18.990	68029.000	15.170	42838.000	13.560	31254.000	18.300	56691.000	14.630	41344.000
1.780	5443.000	1.450	5201.000	1.370	3864.000	1.100	2532.000	1.290	3984.000	1.130	3202.000
18.330	55939.000	14.780	52928.000	16.910	47763.000	16.770	38647.000	15.310	47450.000	16.540	46710.000
0.000	0.000	3.320	11898.000	7.610	21484.000	0.000	0.000	5.540	17160.000	6.420	18147.000
0.300	902.000	0.320	1135.000	0.230	641.000	0.180	406.000	0.290	906.000	0.200	562.000
0.130	391.000	0.080	282.000	0.130	379.000	0.130	292.000	0.140	428.000	0.190	542.000
1.640	5002.000	1.960	7022.000	1.310	3708.000	1.070	2465.000	1.700	5253.000	1.070	3024.000
20.400	62234.000	21.910	78466.000	27.560	77839.000	19.930	47253.000	24.270	75181.000	25.550	72187.000
0.060	177.000	0.100	352.000	0.000	0.000	0.050	116.000	0.080	242.000	0.040	116.000
0.160	503.000	0.230	810.000	0.160	441.000	0.080	185.000	0.300	931.000	0.110	302.000
0.020	74.000	0.010	43.000	0.030	93.000	0.010	24.000	0.000	0.000	0.000	0.000
0.020	66.000	0.030	103.000	0.000	0.000	0.000	0.000	0.020	50.000	0.000	0.000
0.000	0.000	0.000	0.000	0.010	29.000	0.000	0.000	0.000	0.000	0.000	0.000
48.130	146916.000	47.320	169452.000	53.790	151919.000	45.930	107166.000	51.760	160306.000	54.980	155316.000

SP-TE-M-1-3		SP-TE-M-2-1		SP-TE-M-3-1		SP-TE-F-1-4		SP-TE-F-1-5		SP-TE-F-3-3	
Genome_proportion[%]	Nreads	Genome_proportion[%]	Nreads	Genome_proportion[%]	Nreads	Genome_proportion[%]	Nreads	Genome_proportion[%]	Nreads	Genome_proportion[%]	Nreads
13.200	22524.000	3.710	11292.000	5.930	19310.000	8.550	17941.000	8.460	20011.000	11.510	31531.000
0.000	0.000	0.390	1177.000	0.500	1625.000	1.180	2472.000	1.110	2622.000	0.000	0.000
7.400	12633.000	1.440	4378.000	0.000	0.000	3.620	7598.000	4.060	9601.000	0.000	0.000
0.180	313.000	0.080	232.000	0.070	214.000	0.190	396.000	0.200	464.000	0.160	438.000
7.580	12946.000	1.910	5787.000	0.570	1839.000	4.990	10466.000	5.370	12687.000	0.160	438.000
0.360	617.000	0.230	691.000	0.290	928.000	0.340	715.000	0.380	898.000	0.230	640.000
0.000	0.000	0.010	40.000	0.000	0.000	0.390	827.000	0.000	0.000	0.000	0.000
0.220	367.000	0.180	563.000	0.160	515.000	0.280	586.000	0.270	630.000	0.210	576.000
10.260	17497.000	21.040	64077.000	19.870	64645.000	12.860	26989.000	12.680	29999.000	16.770	45941.000
0.000	0.000	0.000	0.000	0.030	106.000	0.000	0.000	0.000	0.000	0.000	0.000
0.070	115.000	0.700	2123.000	0.770	2494.000	0.100	209.000	0.120	283.000	0.370	1008.000
0.020	31.000	0.310	942.000	0.320	1055.000	0.060	125.000	0.070	157.000	0.270	738.000
0.000	0.000	0.010	31.000	0.030	104.000	0.000	0.000	0.000	0.000	0.000	0.000
10.570	18010.000	22.240	67736.000	21.180	68919.000	13.300	27909.000	13.140	31069.000	17.620	48263.000
0.960	1637.000	1.140	3473.000	1.640	5331.000	1.220	2554.000	1.170	2762.000	1.230	3375.000
15.420	26311.000	18.380	55993.000	17.040	55462.000	17.020	35723.000	17.230	40769.000	16.060	43999.000
0.000	0.000	4.630	14089.000	4.100	13345.000	6.200	13006.000	6.650	15737.000	6.230	17057.000
0.060	101.000	0.360	1109.000	0.360	1168.000	0.160	333.000	0.200	481.000	0.230	623.000
0.110	186.000	0.030	99.000	0.000	0.000	0.130	264.000	0.130	310.000	0.100	271.000
0.570	977.000	1.990	6047.000	2.050	6676.000	0.890	1865.000	1.150	2715.000	1.460	3995.000
17.260	30107.000	26.530	80810.000	25.190	81982.000	25.620	53745.000	26.530	62774.000	25.310	69320.000
0.000	0.000	0.090	275.000	0.080	268.000	0.000	0.000	0.000	0.000	0.050	127.000
0.000	0.000	0.270	835.000	0.260	846.000	0.080	178.000	0.050	111.000	0.150	417.000
0.000	0.000	0.030	79.000	0.020	65.000	0.000	0.000	0.000	0.000	0.020	46.000
0.000	0.000	0.040	112.000	0.020	55.000	0.000	0.000	0.000	0.000	0.020	58.000
0.000	0.000	0.000	0.000	0.000	0.000	0.000	0.000	0.000	0.000	0.000	0.000
48.970	84204.000	55.060	167657.000	53.540	174212.000	53.270	111781.00	53.930	127550.000	55.070	150840.000

Supplementary Table 4 Sequence composition of superclusters derived from the comparative analysis repetitive DNA sequence from spinach and sugar beet species.

		Beta_vulgaris	SP-TU-M			SP-TU-F		
			PI647862-M	PI604792-M	PI494751-M	PI647862-F	PI604792-F	PI494751-F
Repeats	Lineage/class	Number of reads	Number of reads	Number of reads	Number of reads	Number of reads	Number of reads	Number of reads
Ty1/Copia	Ale	398	560	618	358	312	546	381
	Angela	1613	64177	54054	38568	83012	67318	40503
	Bianca	936	0	30	0	93	47	0
	SIRE	15554	2320	1256	398	2420	1006	189
	TAR	1303	943	600	443	1122	1138	87
	Tork	858	0	66	24	0	0	47
Ty3/Gypsy	Athila	871	2540	2123	854	4320	2311	1288
	Ogre	0	37680	39588	35826	35553	38488	37587
	Retand	3143	0	4049	5614	0	4232	4281
	CRM	3033	1194	986	296	1221	1267	623
	Galadriel	0	236	71	0	62	0	189
	Tekay	6622	3674	3052	2268	3679	3438	2314
	LINE	0	258	0	0	179	150	119
	Helitron	114	0	0	0	0	0	0
DNA transposons	EnSpm_CACTA	3499	374	351	161	239	458	298
	MuDR_Mutator	257	97	33	0	40	69	0
	Tc1_Mariner	0	174	97	38	104	122	0
Tandem repeats	rDNA	7760	6945	2570	6424	1833	1914	5979
	Satellite	997	523	224	250	412	222	262

SP-OL-M			SP-OL-F			SP-TE-M			SP-TE-F	
PI527332-M	PI6644498-M	PI478393-M	PI527332-F	PI664498-F	PI478393-F	PI647861-M	PI677114-M	PI608712-M	PI647861-F	PI608712-F
Number of reads	Number of reads	Number of reads	Number of reads	Number of reads	Number of reads	Number of reads	Number of reads	Number of reads	Number of reads	Number of reads
648	498	449	332	589	452	189	563	515	518	576
50682	63268	40364	29017	53421	38847	17417	63316	62650	22125	45369
0	45	0	0	0	0	0	0	71	0	0
590	2309	302	200	461	223	50	779	1474	115	714
352	997	508	125	644	448	31	804	1018	103	601
0	43	37	0	30	34	0	31	64	0	0
2312	2419	1188	1058	2305	2006	558	2341	3317	1156	1809
41877	38255	40657	32655	40643	39692	22736	40384	39721	32418	39395
0	4025	7389	0	5411	6577	0	4618	4475	3884	5744
902	1135	598	196	906	320	42	1109	1168	294	538
170	282	266	227	384	488	116	59	0	239	239
2877	3435	1575	983	2986	1259	432	3448	3802	1333	2381
177	313	0	93	0	116	0	111	205	0	127
0	0	0	0	0	0	0	0	0	0	0
279	489	309	98	366	213	0	406	495	71	181
43	43	34	24	0	0	0	79	65	0	46
66	103	0	0	50	0	0	112	55	0	58
11256	5098	12924	10067	330	19254	12946	3690	1839	11577	438
245	265	262	322	248	236	331	233	266	295	180

Supplementary Table 5 Satellites identified by RepeatExplorer and TAREAN analyses (Only repeats with high confidence were analyzed and listed)**.**

Species	Accession	SatDNA Family	Monomer Size (nt)	Abundance (%)
B. vulgaris		BvuSat01-59-s	59	0.018
		BvuSat02-141-s	141	0.037
		BvuSat03-141-s	141	0.041
		BvuSat04-159-s	159	0.300
		BvuSat05-329-s	329	0.290
		BvuSat06-358-s	358	0.210
		BvuSat07-358-s	358	0.200
S. turkestanica	PI647862-M	SpSat01-21	21	0.042
		SpSat02-52	52	0.760
		SpSat03-179	179	0.052
		StuSat01-158-s	158	0.010
	PI604792-M	SpSat01-21	21	0.074
		SpSat03-179	179	0.070
	PI494751-M	SpSat01-21	21	0.100
		SpSat02-52	52	3.000
		SpSat03-179	179	0.089
	PI647862-F	SpSat01-21	21	0.027
		SpSat02-52	52	0.470
		StuSat01-158-s	158	0.013
	PI604792-F	SpSat02-52	52	0.920
		SpSat03-179	179	0.051
	PI494751-F	SpSat01-21	21	0.100
		SpSat02-52	52	3.000
		SpSat03-179	179	0.082
S. oleracea	PI527332-M	SpSat01-21	21	0.065
		SpSat02-52	52	1.400
		SpSat03-179	179	0.080
		SpSat04-324	324	0.015
	PI664498-M	SpSat01-21	21	0.039
		SpSat02-52	52	0.670
		SpSat03-179	179	0.054
		SpSat04-324	324	0.020
	PI478393-M	SpSat01-21	21	0.093
		SpSat02-52	52	1.800
		SpSat03-179	179	0.067
		SpSat04-324	324	0.011
	PI527332-F	SpSat01-21	21	0.140
		SpSat02-52	52	3.300
		SpSat04-324	324	0.013
	PI664498-F	SpSat01-21	21	0.061
		SpSat02-52	52	1.400
		SpSat03-179	179	0.080

		SpSat05-141	141	0.018
		SolSat01-171-s	171	0.010
	PI478393-F	SpSat01-21	21	0.079
		SpSat02-52	52	1.900
		SpSat03-179	179	0.084
S. tetrandra	PI647861-M	SpSat01-21	21	0.190
		SpSat02-52	52	5.200
		SpSat03-179	179	0.072
		SteSat01-99-s	99	0.026
	PI677114-M	SpSat02-52	52	1.200
		SpSat03-179	179	0.072
	PI608712-M	SpSat01-21	21	0.017
		SpSat02-52	52	1.100
		SpSat03-179	179	0.060
	PI647861-F	SpSat01-21	21	0.130
		SpSat02-52	52	3.500
		SpSat03-179	179	0.087
		SpSat04-324	324	0.021
		SpSat05-141	141	0.016
	PI608712-F	SpSat01-21	21	0.062
		SpSat02-52	52	2.000

Supplementary Table 6 Tandem repeats monomer consensus sequences.

> BvuSat01-59-s monomer consensus sequences (59 bp)

ACCCAGGGGCCAATGAGCCCCAGGTAACTCGAGGAAGTTAAGAGACCTCGCTCGAGGTT

> BvuSat02-141-s monomer consensus sequences (141 bp)

TTTGCGGGGTTTAACCTCAACAGCACAGAATTGCACAGGTTAGTTCTGGATCTTTCGAATTCACGTATTTTATACTG
TACTAGCTCGAAATTTTGGCACATTATCCAACTCAAACATCTTCAATCCGCGCTTTTGGGGGAT

> BvuSat03-141-s monomer consensus sequences (141 bp)

CGCCCCAAATTGGAGTTGTCGTGTCTTAACGTGTCCAATCAAGACCTAGAAAGCTTAAGTGCATAAAATATGACAT
GATCGAGCTTTATAACCGTGTAATAGGTTGAGTTTGTAGAAGTTAGGCGCGAAAACCCCCTAAAA

> BvuSat04-159-s monomer consensus sequences (159 bp)

ACATTTCAGGCTTTTGAATCGCTCAATTCTGATGTCCGAGCTAGGAGTTAGGCCCAAATTACGATTTTGGGCCAAG
ATGAGCAGGAAACAGACGCGAAAAATCGGACTTCAACGAATTCAGCTTCTGAGCTCACGAACCCCTTAGAAATGG
TAAAAAAA

> BvuSat05-329-s monomer consensus sequences (329 bp)

AGGTTAATGAGAGATCCTCTAGTTCTCGAGCTAACAACAAAGCCTATTCAATATAAAGATTCAATCTTTATGCCCT
ATTTCATATAAAGATCGAATCTTTCAACAAGTACAAGTGAGATTCGGGTGTTTTGAGACTTGTTATTGATTTTACTA
TCCACAAACACATCATTTGCTTTATAATTCTTTTTTTTAGTTGTTGAATGATATAGGGGTCCTATTATGTTTATGAGT
CTAATTCCCTTAAATCAGGTGTGTGTGTGTTTTAATTTAATAATTCGAGTGCATTGCATTGAATATACAAATTGAGG
TTTGTGTGCATCTCCTAATAG

> BvuSat06-358-s monomer consensus sequences (358 bp)

ACTCTTTAGTTTGTATAAAAGTTTTTAGGAGGCTCAATCACTTGGCCCCATGTGCGGACTCACAATAAAGTCTCTA
AAGTATTTTAGTTTGATTCCTACCGAGAAGACCAGTGGCTCATCTGAATCCCAAGGACACATAACTAAAGATCTGC
CAGCTTCAAGGAAGACTACGGTAAAAAGATTTCTAAAGTTGTTAAGAAATTTTAGAGATACAAGCCACTGGGGGC
CATGTGCGGACACTCCGTAAAAAGCAATAATGAATTTTGATAGGATTTTTCCGTTGTGCTTTGGAAACCTTCTGAA
ACCGAAAGTGATAAACCCCGGGAAAACCAGTTTACACGAAATGTCACGGTAAAAT

> BvuSat07-358-s monomer consensus sequences (358 bp)

GCACTGTAAAGCACATTTGACCAAAAGGGCCCCAAATAGTGAAAGCCAAAGTCTTCCAAAGGTTTCGTGTTGCCT
TTTTAGGATAGTTTTAAGTAATCACGAAAAATGCCTCACAGGCGTGTACCGGGGGTCACCGAACATAGAGATTTTA
AAGAATTGTTGAAATCTTTAGAAAAATGGCATCAGAAGGAACTTCGACCGTCTAGAAATCAATACACAGGAACCC
TAAGTCTACTCGGTGACCAGAAGAGCCATCCTTAGTTTGATTTTATGAAATCTCTGAAATAACACTCAGGCGTGTA
CCCCGGTTCACTAACTCGGAGGATTTTTGAAAATATGTTTGATTTCTCATAAAATG

> SpSat01-21 monomer consensus sequences (21 bp)

GCTATCGGCACCCGCCAACTA

> SpSat02-52 monomer consensus sequences (52 bp)

CAAGAAAGGTAAGCAAGGGCACGACATGGCCCAAGCAAGGCAAGGCATGACC

> SpSat03-179 monomer consensus sequences (179 bp)

AGGCAAAGGAGATGGTTTTAAGCACCTAATAGGGCCTTAAGGGACCCATGGGGTATGTGGTAAGCGCTCGGACCT
CTAACTTTGACGGTTTCGAAAAACGCCCAGGCTCAAAGGTCCCGGTATGGCATCGACTCGACAACTCGTGTTTTTT
CAGAAAGTGGGCTGAATTAGCCTTTAGG

> SpSat04-324 monomer consensus sequences (324 bp)

TAAAAAAAAACCACAAAACGGGTCAAATTTGGTCGAAAAGTGTGCATTATAGGTGGGCGATTTTGGCTAATTTTT
ACATTTTAAGAAACTTGAAACGTTAGAGGTCCGGGCGCTTCCCACATACCCTTTGGGTCCCCAGGGGTCATATTGG
GTGCTTAGCAGCATCTCCCCGGCCTCCTAAATGGCATTTAAAGGGACTTCGAAAAAACGCGCGTTGTGAAATAAA
TGGAATTCCGGGGCCTTAAGGGACCAATAAGTTGGTTTTTTCCCGACCAAACCTTATTATATGACCATATTATACA
TTCCCCAAAATTTGAGGAGATC

> SpSat05-141 monomer consensus sequences (141 bp)

CTCGTCATGACTTGCAAGCCGTCATCCTAATGCTCTCGTCATGACTCATGCCTAGCCACATGTTGGAGCTTAAAAT
CGCTCACAACCATGTGCCCTCGTCGCAACTTATGACACGTTACCTCTCGTCATAAGTCTATTGCT

> StuSat01-158-s monomer consensus sequences (158 bp)

CCTATTTTTTCACCCACTATTAGGCCGTGCATTCAGAAATTCGAGAATTAGAGTTTCAATTTTCCCTAAAATGGGTG
CCCTAAAACGAAAGCCCTGAATTATGACCCCATAACTTGGTGCTCGGGAATGATTTTGACCGGATTCTTTTTTAAA
AGTTG

> SolSat01-171-s monomer consensus sequences (171 bp)

GGGTGAGAATGAGTCTTTGAAACATAAAACTAAGTGTATTAAACATGGAAAGACTTTAAAATACTCATTTAAGTT
CATTAGTTGAAAGTTGGGACCGAAATTAGCCATTTTAGTCAAAATGTGACCGATAATGAACTAACGAGGCTTAAA
TGTGCATAACTTCACCAAAAT

> SteSat01-99-s monomer consensus sequences (99 bp)

AACCTCGCTCTTCAAATCCGGAGATCTGTTTAAGAACGTGCTTCTAAAATCGGCTCGGATTGTAACATCGGTTCGA
GTTGTAATCTCGGAGCGATTTTT

Supplementary Table 7 The results of SolSat01-171-s blasted against the sex determination region (SDR) in chromosome 1.

Query id	Subject id	Percentage of identical matches	Start of alignment in subject	End of alignment in subject	E-value
SolSat01-171-s	chr1	98.83	8643644	8643474	8.49E-82
SolSat01-171-s	chr1	98.246	547791	547621	3.61E-80
SolSat01-171-s	chr1	98.246	547962	547792	3.61E-80
SolSat01-171-s	chr1	98.225	549323	549155	4.40E-79
SolSat01-171-s	chr1	98.225	8641257	8641089	4.40E-79
SolSat01-171-s	chr1	98.225	8641597	8641429	4.40E-79
SolSat01-171-s	chr1	97.661	1423340	1423510	5.36E-78
SolSat01-171-s	chr1	97.633	8637785	8637617	5.36E-78
SolSat01-171-s	chr1	97.059	16008076	16008245	6.53E-77
SolSat01-171-s	chr1	96.491	8638296	8638126	2.28E-76
SolSat01-171-s	chr1	96.491	8638978	8638808	2.28E-76
SolSat01-171-s	chr1	97.041	8645175	8645007	2.28E-76
SolSat01-171-s	chr1	96.491	12726415	12726245	2.28E-76
SolSat01-171-s	chr1	96.491	12731309	12731139	2.28E-76
SolSat01-171-s	chr1	97.041	12733351	12733183	2.28E-76
SolSat01-171-s	chr1	96.491	16006876	16007046	2.28E-76
SolSat01-171-s	chr1	96.491	8637955	8637786	2.78E-75
SolSat01-171-s	chr1	96.45	8638636	8638468	2.78E-75
SolSat01-171-s	chr1	96.45	8643131	8642963	2.78E-75
SolSat01-171-s	chr1	96.491	12732838	12732669	2.78E-75
SolSat01-171-s	chr1	95.906	1422314	1422484	9.69E-75
SolSat01-171-s	chr1	95.906	1422998	1423168	9.69E-75
SolSat01-171-s	chr1	95.906	8639829	8639659	9.69E-75
SolSat01-171-s	chr1	95.906	8643302	8643132	9.69E-75
SolSat01-171-s	chr1	95.906	8644323	8644153	9.69E-75
SolSat01-171-s	chr1	95.906	8644494	8644324	9.69E-75
SolSat01-171-s	chr1	95.906	8644665	8644495	9.69E-75
SolSat01-171-s	chr1	95.906	8645516	8645346	9.69E-75
SolSat01-171-s	chr1	95.882	12732160	12731991	3.38E-74
SolSat01-171-s	chr1	95.882	12732499	12732330	3.38E-74
SolSat01-171-s	chr1	95.906	548473	548304	1.18E-73
SolSat01-171-s	chr1	95.858	8639658	8639490	1.18E-73
SolSat01-171-s	chr1	95.322	8642108	8641938	1.18E-73
SolSat01-171-s	chr1	95.322	8642279	8642109	1.18E-73
SolSat01-171-s	chr1	95.858	8642619	8642451	1.18E-73
SolSat01-171-s	chr1	95.322	8643473	8643303	1.18E-73
SolSat01-171-s	chr1	95.322	8643815	8643645	1.18E-73
SolSat01-171-s	chr1	95.322	8644152	8643982	1.18E-73
SolSat01-171-s	chr1	95.322	8644836	8644666	1.18E-73
SolSat01-171-s	chr1	95.322	12571335	12571165	1.18E-73
SolSat01-171-s	chr1	95.858	12571676	12571508	1.18E-73
SolSat01-171-s	chr1	95.322	12724369	12724199	1.18E-73

SolSat01-171-s	chr1	95.906	12725733	12725563	1.18E-73
SolSat01-171-s	chr1	95.906	12728370	12728200	1.18E-73
SolSat01-171-s	chr1	95.906	12731820	12731650	1.18E-73
SolSat01-171-s	chr1	95.833	8645852	8645685	4.12E-73
SolSat01-171-s	chr1	95.349	16007047	16007218	4.12E-73
SolSat01-171-s	chr1	95.266	8639318	8639150	1.44E-72
SolSat01-171-s	chr1	95.266	8640340	8640172	1.44E-72
SolSat01-171-s	chr1	95.322	12727648	12727478	1.44E-72
SolSat01-171-s	chr1	95.322	12729092	12728922	1.44E-72
SolSat01-171-s	chr1	94.737	550344	550174	5.02E-72
SolSat01-171-s	chr1	94.737	1422143	1422313	5.02E-72
SolSat01-171-s	chr1	94.737	12725222	12725052	5.02E-72
SolSat01-171-s	chr1	94.737	12727097	12726927	5.02E-72
SolSat01-171-s	chr1	95.294	12732668	12732500	5.02E-72
SolSat01-171-s	chr1	94.152	548303	548133	6.11E-71
SolSat01-171-s	chr1	94.675	548643	548475	6.11E-71
SolSat01-171-s	chr1	94.737	549153	548984	6.11E-71
SolSat01-171-s	chr1	94.152	8640000	8639830	6.11E-71
SolSat01-171-s	chr1	94.675	8640680	8640512	6.11E-71
SolSat01-171-s	chr1	94.737	8646023	8645854	6.11E-71
SolSat01-171-s	chr1	94.152	12724029	12723859	6.11E-71
SolSat01-171-s	chr1	94.152	12724882	12724712	6.11E-71
SolSat01-171-s	chr1	94.152	12726926	12726756	6.11E-71
SolSat01-171-s	chr1	94.152	12727990	12727820	6.11E-71
SolSat01-171-s	chr1	94.152	12729774	12729604	6.11E-71
SolSat01-171-s	chr1	94.152	12730287	12730117	6.11E-71
SolSat01-171-s	chr1	94.152	12730627	12730457	6.11E-71
SolSat01-171-s	chr1	94.186	1422485	1422656	2.13E-70
SolSat01-171-s	chr1	94.186	8642791	8642620	2.13E-70
SolSat01-171-s	chr1	94.737	12723346	12723179	2.13E-70
SolSat01-171-s	chr1	94.643	12725391	12725224	2.13E-70
SolSat01-171-s	chr1	94.643	12726584	12726417	2.13E-70
SolSat01-171-s	chr1	94.643	12731478	12731311	2.13E-70
SolSat01-171-s	chr1	94.706	12732329	12732161	2.13E-70
SolSat01-171-s	chr1	94.152	549493	549324	7.45E-70
SolSat01-171-s	chr1	94.152	8637615	8637446	7.45E-70
SolSat01-171-s	chr1	94.152	8638466	8638297	7.45E-70
SolSat01-171-s	chr1	94.152	8639148	8638979	7.45E-70
SolSat01-171-s	chr1	94.152	8640510	8640341	7.45E-70
SolSat01-171-s	chr1	94.083	8641937	8641769	7.45E-70
SolSat01-171-s	chr1	94.152	8645345	8645176	7.45E-70
SolSat01-171-s	chr1	94.118	550173	550005	2.60E-69
SolSat01-171-s	chr1	93.567	550515	550345	2.60E-69
SolSat01-171-s	chr1	93.567	8638807	8638637	2.60E-69
SolSat01-171-s	chr1	93.567	12571506	12571336	2.60E-69
SolSat01-171-s	chr1	93.567	12727268	12727098	2.60E-69
SolSat01-171-s	chr1	93.567	12728712	12728542	2.60E-69

SolSat01-171-s	chr1	93.567	12729434	12729264	2.60E-69
SolSat01-171-s	chr1	92.982	1422827	1422997	3.17E-68
SolSat01-171-s	chr1	93.567	1423169	1423338	3.17E-68
SolSat01-171-s	chr1	93.491	8638125	8637957	3.17E-68
SolSat01-171-s	chr1	93.567	8640170	8640001	3.17E-68
SolSat01-171-s	chr1	93.567	8642961	8642792	3.17E-68
SolSat01-171-s	chr1	92.982	12572019	12571849	3.17E-68
SolSat01-171-s	chr1	92.982	12725904	12725734	3.17E-68
SolSat01-171-s	chr1	92.982	12726075	12725905	3.17E-68
SolSat01-171-s	chr1	92.982	12728541	12728371	3.17E-68
SolSat01-171-s	chr1	92.982	12729263	12729093	3.17E-68
SolSat01-171-s	chr1	92.982	12730116	12729946	3.17E-68
SolSat01-171-s	chr1	92.982	12730969	12730799	3.17E-68
SolSat01-171-s	chr1	92.982	16007562	16007732	3.17E-68
SolSat01-171-s	chr1	92.941	1421802	1421971	1.11E-67
SolSat01-171-s	chr1	93.452	12729603	12729436	1.11E-67
SolSat01-171-s	chr1	93.452	12730456	12730289	1.11E-67
SolSat01-171-s	chr1	93.023	16007390	16007561	1.11E-67
SolSat01-171-s	chr1	92.982	548132	547963	3.86E-67
SolSat01-171-s	chr1	93.491	8639488	8639321	3.86E-67
SolSat01-171-s	chr1	93.491	8641427	8641260	3.86E-67
SolSat01-171-s	chr1	93.491	8641767	8641600	3.86E-67
SolSat01-171-s	chr1	92.899	16007906	16008074	3.86E-67
SolSat01-171-s	chr1	92.398	8643981	8643816	1.35E-66
SolSat01-171-s	chr1	92.398	8645683	8645517	1.35E-66
SolSat01-171-s	chr1	92.398	12723687	12723517	1.35E-66
SolSat01-171-s	chr1	92.398	12724711	12724541	1.35E-66
SolSat01-171-s	chr1	92.398	16007219	16007389	1.35E-66
SolSat01-171-s	chr1	92.442	12571848	12571677	4.70E-66
SolSat01-171-s	chr1	91.429	16006701	16006875	4.70E-66
SolSat01-171-s	chr1	92.398	548813	548644	1.64E-65
SolSat01-171-s	chr1	92.398	549663	549494	1.64E-65
SolSat01-171-s	chr1	92.398	1422657	1422826	1.64E-65
SolSat01-171-s	chr1	92.398	8641087	8640918	1.64E-65
SolSat01-171-s	chr1	91.813	12723858	12723688	1.64E-65
SolSat01-171-s	chr1	91.813	12724540	12724370	1.64E-65
SolSat01-171-s	chr1	91.813	12727819	12727649	1.64E-65
SolSat01-171-s	chr1	91.813	12729945	12729775	1.64E-65
SolSat01-171-s	chr1	91.813	12730798	12730628	1.64E-65
SolSat01-171-s	chr1	94.904	8640837	8640681	5.73E-65
SolSat01-171-s	chr1	92.262	12731988	12731821	5.73E-65
SolSat01-171-s	chr1	92.398	8645005	8644837	2.00E-64
SolSat01-171-s	chr1	91.813	12731649	12731479	2.00E-64
SolSat01-171-s	chr1	91.667	12725051	12724884	6.98E-64
SolSat01-171-s	chr1	91.667	12726244	12726077	6.98E-64
SolSat01-171-s	chr1	91.667	12731138	12730971	6.98E-64
SolSat01-171-s	chr1	91.228	16007733	16007903	6.98E-64

SolSat01-171-s	chr1	91.228	8642449	8642280	8.50E-63
SolSat01-171-s	chr1	91.071	12724198	12724031	2.97E-62
SolSat01-171-s	chr1	90.643	548983	548814	1.04E-61
SolSat01-171-s	chr1	90.643	12723516	12723347	1.04E-61
SolSat01-171-s	chr1	90.476	549831	549664	3.61E-61

Supplementary Figure 1 Topological layout of satellite DNAs. a, b, c and d indicate topology layouts of SpSat01-21, SpSat02-52, SpSat03-179 and SpSat04-324, respectively.

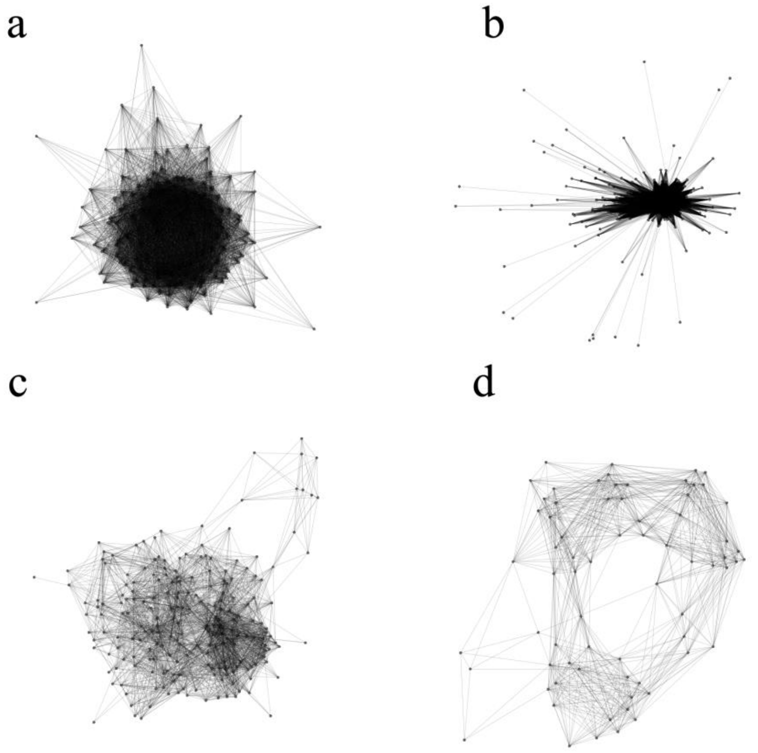

Supplementary Figure 2 Consensus sequence of satellite DNAs. a, b, c and d show consensus sequences of SpSat01-21, SpSat02-52, SpSat03-179 and SpSat04-324, respectively.

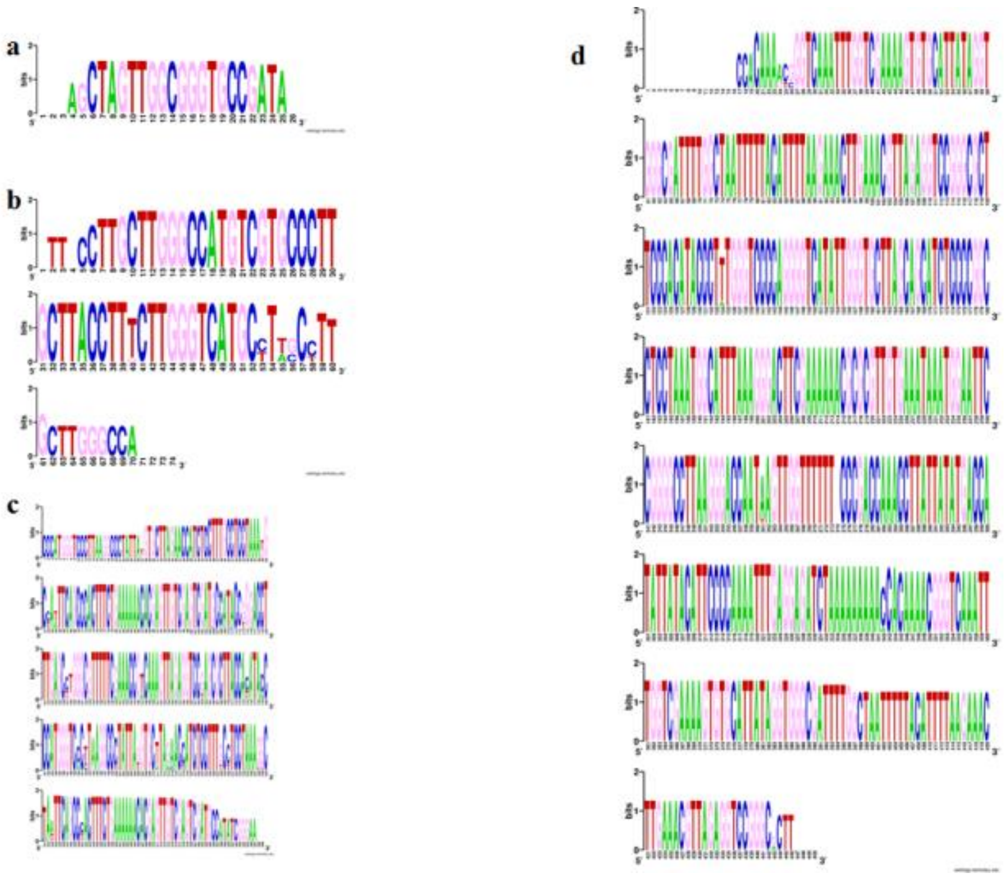

Supplement: Supplementary file 1 [file Table_1.pdf]
